# Chemical and Physical Modification of Lignin for Green Polymeric Composite Materials

**DOI:** 10.3390/ma16010016

**Published:** 2022-12-20

**Authors:** Karolina Komisarz, Tomasz M. Majka, Krzysztof Pielichowski

**Affiliations:** Department of Chemistry and Technology of Polymers, Faculty of Chemical Engineering and Technology, Cracow University of Technology, ul. Warszawska 24, 31-155 Kraków, Poland

**Keywords:** lignin, chemical modification, physical modification, biopolymers, biocomposites

## Abstract

Lignin, a valuable polymer of natural origin, displays numerous desired intrinsic properties; however, modification processes leading to the value-added products suitable for composite materials’ applications are in demand. Chemical modification routes involve mostly reactions with hydroxyl groups present in the structure of lignin, but other paths, such as copolymerization or grafting, are also utilized. On the other hand, physical techniques, such as irradiation, freeze-drying, and sorption, to enhance the surface properties of lignin and the resulting composite materials, are developed. Various kinds of chemically or physically modified lignin are discussed in this review and their effects on the properties of polymeric (bio)materials are presented. Lignin-induced enhancements in green polymer composites, such as better dimensional stability, improved hydrophobicity, and improved mechanical properties, along with biocompatibility and non-cytotoxicity, have been presented. This review addresses the challenges connected with the efficient modification of lignin, which depends on polymer origin and the modification conditions. Finally, future outlooks on modified lignins as useful materials on their own and as prospective biofillers for environmentally friendly polymeric materials are presented.

## 1. Nomenclature and Abbreviations

This review focuses on physical and chemical modification of lignin with various agents. Table 1 contains all the abbreviations used in the review with their respective explanations.

## 2. Background

**Figure 1:**
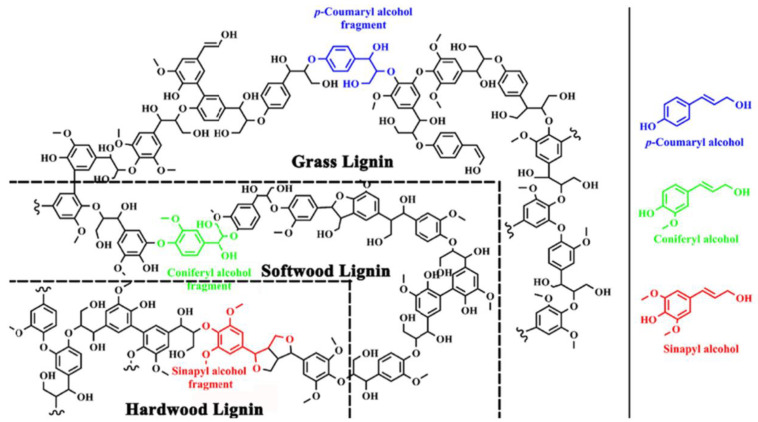
Precursor monolignols and lignin structure resulting from the plant source. Reproduced from [13], with the permission of Elsevier.

Lignocellulosic biomass constitutes the most abundant source of renewable biomass, which consists mainly of three biopolymeric elements—cellulose, hemicelluloses, and lignin, which form complex, interpenetrating structures in plant organisms [1,2,3,4,5]. Both cellulose and hemicellulose fractions are comprised of polysaccharides [1,2,3,6,7,8], whereas lignin is an aromatic macromolecule composed of three phenylpropane units, originating from three aromatic alcohol precursor components (monolignols), namely p-coumaryl, coniferyl, and sinapyl alcohols (Figure 1) [9]. Structures originating from the mentioned monolignols are p-hydroxyphenyl (H, from coumaryl alcohol), guaiacyl (G, from coniferyl alcohol), and syringyl (S, from sinapyl alcohol), which are crosslinked mainly by β-O-4 and α-O-4 ether, β-β resinol, and 5-5′ biphenyl bonds [9,10,11]. The amount and composition of lignin vary between different plant species, being the source of lignocellulosic biomass, as well as its growing age, which is presented in Table 2 [10,11,12].

**Figure 2:**
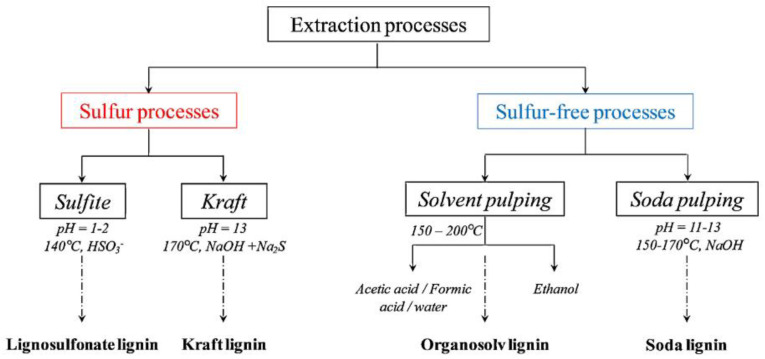
The processes of lignin extraction from lignocellulosic biomass and the respective types of technical lignin. Reproduced from [9], with the permission of Elsevier.

There are four main methodologies used for the extraction of lignin from biomass: kraft, sulfite, soda pulping, and solvent pulping (Organosolv) processes. Both kraft and sulfite processes use sulfur-based chemicals to facilitate lignin extraction, whereas soda and Organosolv processes are sulfur-free. Figure 2 depicts the possible methods of obtaining technical lignin from cellulosic biomass.

In industrial paper-making processes, the Organosolv technique is a pulping technique that uses an organic solvent to solubilize lignin. The process was invented by Kleinert in 1968 as an environmentally benign alternative to kraft pulping. In particular, the ability to obtain relatively high-quality lignin adds value to a process stream otherwise considered waste. Organosolv solvents are easily recovered by distillation, leading to less water pollution and elimination of the odor usually associated with kraft pulping. This is the reason why Organosolv modification is the most popular type of modification and Organosolv lignin is one of the pure types of lignin that is used in chemical reactions [14].

The choice of method determines the properties of the resultant technical lignin; moreover, not all methods are applicable to different plant sources. In Table 3, typical properties of various technical lignin are presented.

Sulfur lignin, which comprises lignosulfonates and kraft lignin, is a by-product of the paper and pulping industry utilizing cellulose extraction from lignocellulosic biomass. The kraft method involves a high-temperature delignification process in the presence of sodium hydroxide and sodium sulfide, which leads to the cleavage of ether linkages and solubilization of lignin that can be isolated from the alkaline solution by precipitation using sulfuric acid [11]. Kraft lignin contains about 1–2% of sulfur, a high number of condensed structures, and phenolic hydroxyl groups [9]. Lignosulfonates are a product of the sulfite process, in which lignocellulosic biomass is cooked with the aqueous solution of sulfur dioxide, and the sulfites are generated by the addition of the chosen base: ammonium, calcium, magnesium, or sodium. Introduction of polar sulfonic groups into the lignin backbone leads to the increase of solubility of lignosulfonate in water, and to precipitate it, the addition of sulfuric acid is needed. The sulfur content of lignosulfonates varies between 4% and 8% and is connected to the presence of the sulfonic acid groups. They are used industrially as binders, dispersing agents, surfactants, adhesives, and cement additives. The presence of the cations remaining from the pulping process impacts the reactivity of lignosulfonates—ammonium-based materials display the highest reactivity, whereas lignosulfonates obtained using a calcium base exhibit the lowest reactivity [9,11].

As for sulfur-free lignins, they are characterized by a lower molar mass and dispersity than their sulfur-based counterparts. The structure of Organosolv and soda lignin is more resemblant of the native lignin and can act as an attractive, renewable source of aromatic compounds [15]. Soda lignin is obtained by extraction from non-wood plant sources such as grass, straw, or sugarcane bagasse. The process resembles the kraft process, as biomass is subjected to the action of an aqueous sodium hydroxide solution with a small addition of hydroquinone as a catalyst. The resultant technical product is hard to recover but does not contain sulfur [11]. Organosolv lignin is considered to be one with the least amount of impurities and is highly hydrophobic, which enables its dissolution in various organic solvents. The Organosolv process of treatment of lignocellulosic biomass involves the usage of organic solvents as delignifying agents, the most commonly used being ethanol, methanol, acetic, and formic acid, which are mixed with water. The recovery of the lignin is achieved by the evaporation of organic solvent or by precipitation. The Organosolv process preserves the natural structure of lignin, however, due to low molecular mass, extensive corrosion of equipment, and possible condensation reactions, it is not as widely used as kraft or soda pulping [11].

Lignin, in contrast to cellulose and hemicelluloses, does not exhibit a regular structure. This fact is connected to both internal (1) and external (2) factors: (1) lignin botanic source, age of the plant, biosynthetic pathway, and function, and (2) geological area, soil type, and procedure of extraction. Unmodified lignin may be utilized without further modification as a fuel, adsorbent material, or precursor for carbon-based materials, but its structure allows for participation in various types of chemical reactions [13].

Although lignin can be utilized without further modification as an antioxidant, a UV stabilizer, or a flame retardant [16,17,18,19], it can only be incorporated in small amounts because of its rather poor miscibility with polymer matrices [20,21,22,23], which points towards the development of new methods of modification as a way to obtain better compatibility with polymer matrices and to impart desired properties to the modified material.

In recent years, an observable trend has been noticed in the development and growth of the polymer materials market. At the same time, the depleting resources of a petrochemical nature have shifted the focus of many researchers towards renewable resources as a potential alternative source of many important chemicals as well as a way of reducing greenhouse gases [24]. Following this trend, much interest has been paid to lignocellulosic biomass and its three main constituents: cellulose, hemicelluloses, and lignin, which can be utilized not only in the paper and wood industry but as precursors for alcohols, sugars [25], phenolic derivatives, and as natural fillers for polymeric composites [26].

The latter topic dealing with lignin modification/functionalization concentrated mainly on the utilization of chemical processes. As it was mentioned earlier, although it is possible to utilize technical lignin without further modification, modification is a necessary step to obtain value-added products. As for the main chemical pathways in lignin modification, it is possible to identify three main routes:

(a) Fragmentation or depolymerization of lignin to yield carbon materials and chemicals rich in aromatic structures [27].
(b) Chemical modification by reactions of lignin hydroxyl groups with various agents.
(c) Creation of new chemically active sites.

This review concentrates on the methods of lignin modification which can be utilized for the application in various polymeric composites, involving both reactions of the hydroxyl groups present in lignin as well as the introduction of new chemically active sites to its structure. The wide range of possible reactions of hydroxyl groups, including esterification [28], alkylation [29], arylation [30], epoxidation [31], etherification [32], and amination [33], among many others, allow for versatility and possible applications as a filler or functional additive for many polymeric systems and matrices. Aside from the mentioned reactions, other ways of chemical enhancement of the properties of lignin were reported, including copolymerization, silylation, and grafting. This review also includes some physical approaches to lignin modification, leading to the different surface properties of the lignin macromolecules. Finally, a summary of the modifying agents used in various types of modification, as well as the description of the properties’ enhancements, have been presented.

## 3. Types of Modifications and Modifiers

In Table 4, the modification methods of lignin and chemical agents are presented.

Most of the methods employed for the modification of lignin are of chemical origin. A variety of compounds for obtaining ester-derivatives of lignin have been presented, accompanied by hydroxymethylated and amine-based lignin derivatives.

All of the lignin modifications presented in this review were used in various polymeric matrices, including conventional polymers, such as low- and high-density polyethylene (LDPE, HDPE), polypropylene (PP), polystyrene (PS), poly(ethylene terephthalate) (PET), polyurethane systems (PU), natural and butadiene rubber (NR, BR), as well as poly(butylene adipate-co-terephthalate) (PBAT), poly(butylene succinate) (PBS), polyhydroxyalkanoates (PHA), and polylactide (PLA).

The reported methods of physical modification focus on utilizing the physical processes to impart new properties to lignin, not necessarily involving the use of chemicals, and these methods include freeze-drying, UV and gamma irradiation, plasma treatment, ultrasonic homogenization, and sorption of compounds onto the surface of lignin [34].

Lignin modification via esterification mainly focuses on the application of carboxylic acids and their derivatives as the modifying agents. The compounds from this group described in the literature comprise the following examples:

- Acyl chlorides: 10-undecenoyl chloride, oleoyl chloride [35].
- Carboxylic anhydrides: acetic [28,36,37,38,39,40], propionic [29,39], butyric [29,39,41,42,43], isobutyric, crotonic [29], methacrylic [29,44], succinic [45], maleic [46,47], and phthalic anhydride [48].
- Carboxylic acids: oleic, butyric, lactic [49], and acrylic acid [50].
- Lactones: butyrolactone [49] and γ-valerolactone [43].

Other compounds described in the esterification processes include tung oil, n-butyl methacrylate [50], phosphorus chloride [51,52,53,54], triethylamine [53,54], diphenyl phosphoryl chloride [55], and 3-chloro-2-hydroxypropyltrimethyl ammonium chloride [56,57].

In the case of alkylation and arylation of lignin, the employed modifiers are chlorinated hydrocarbons, such as dichloromethane and chlorobenzene [30], heterocyclic hydrocarbons such as tetrahydrofuran [58,59], and carboxylic acids and derivatives such as butyrolactone [58] and lactic acid [59].

Epoxidation of lignin involves the reaction of phenolic hydroxyl groups with epichlorohydrin to obtain functional additives for epoxy and polyurethane systems [31,60,61].

The reports involving the etherification of lignin to yield functional fillers and additives focus mainly on hydroxymethylation using formaldehyde [53,61,62,63,64,65,66], although other aldehydes and related compounds are also employed:

- Propylene oxide [32,66,67].
- Glyoxal and glutaraldehyde [66].

Copolymerization reactions involve the use of different compounds to enhance the intrinsic properties of lignin. The examples are N-methylaniline [68], ε-caprolactone [69,70], L-lactide [69,71], and butyrolactone [70,72].

Amination of lignin via the Mannich reaction mechanism employs the reaction of lignin with amine compounds in the presence of formaldehyde. Different amines were employed in such processes, such as dimethylamine [73], methylamine [74], poly(ethylene imine) [75], diethylenetriamine [76,77,78], isophorone diamine [79], urea [80], and glycine [81].

Silylating agents used as chemical modifiers of lignin properties are, e.g., silica [82,83,84], (3-aminopropyl)triethoxysilane [85], allyltrimethoxysilane [40], tert-butyldimethylsilyl chloride [86], and γ-divinyl-3-aminopropyltriethoxysilane [87].

Other reported types of modification included in the review utilize dimethyl(methacryloyl)methyl phosphonate [88], methyl methacrylate [88,89,90], ethyl and benzyl methacrylates [89,90], n-butyl methacrylate [90], poly(ethylene imine) [91,92], diethyl phosphite [91,92], and dihydroxybenzenes—catechol, resorcinol, and hydroquinone [93].

## 4. Chemical Modification

### 4.1. Esterification

**Figure 3:**
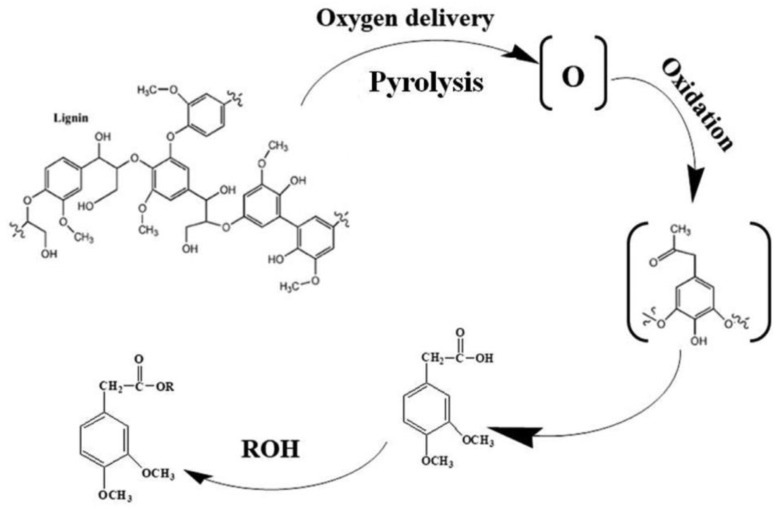
Esterification of lignin. Reproduced from [94], with the permission of Elsevier.

Esterification of lignin in the reaction with carboxylic acids, anhydrides, and acid chlorides is the most commonly employed method of chemical modification of this polymer (Figure 3). Lignin modification by esterification reaction with carboxylic acids and derivatives yields numerous property changes, such as:

- Better UV absorption [35,47],
- Changed thermal stability [35,37,43,44],
- Better compatibility with the matrix [35,36,39,46],
- Improved mechanical properties [35,36,37,38,45,46,48],
- Better dimensional stability [28],
- Improved hydrophobicity [28,37],
- Resistance to microbial decay [28],
- Changed mechanical properties [28,41].

Xing et al. [35] reported the esterification of soda lignin with 10-undecenoyl chloride and oleoyl chloride, carried out at 65 °C for 48 h without additional use of solvents or catalysts. At the end of the reaction, methanol was added to the reaction to quench unreacted chlorides. After the removal of the solvent under vacuum, the products were dried in the vacuum oven for 24 h. Composites of poly(butylene adipate-co-terephthalate) (PBAT) with 10 and 20 wt.% of unmodified and modified lignin were obtained via extrusion in a twin-screw micro-compounder with a nitrogen purge. The extruded strands were then cut into pieces and subjected to pressure molding into thin films. The obtained films were subjected to UV irradiation to assess their UV-absorption capacity. Undecenoyl-treated lignin was found to have a higher UV-absorption capacity, while oleoyl-modified lignin showed better thermal stability. Modification of lignin with undecenoyl chloride resulted in better compatibility of the filler with the PBAT matrix, improved mechanical properties of the composites, and only slightly decreased thermal stability [35].

Acetylation of lignin is another commonly employed method of lignin modification via esterification. Nevarez et al. [36] subjected kraft, Organosolv, and hydrolytic lignin to a reaction with acetic anhydride with 4-dimethyl amino pyridine as a catalyst. Acetylated kraft lignin was precipitated using the addition of ethyl ether, while in the case of modified Organosolv and hydrolytic lignin, a cold mixture of water and methanol was used to obtain the precipitate. Residues were washed with a mixture of water and methanol and dried. Then, films of cellulose triacetate (CTA) composite with 1 wt.% of modified and unmodified lignin were obtained via the method of solvent casting, using methylene chloride as a solvent. Results of the reported study show that acetylated lignin exhibits good homogeneity with the matrix, regardless of the lignin origin. Mechanical properties of CTA composites were generally improved, with only a few exceptions. CTA-based membranes can be applied in water purification processes, wherein incorporation of lignin adds to the resistance to biofouling, owing to the antibacterial properties of lignin [36]. Lignin acetylation was also described in the work of Pradhan et al. [28], where kraft lignin was reacted with acetic or propionic anhydride. Esterified lignin was then blended with PLA, maintaining the loading of lignin at 10 wt.%. Results show that composites exhibit better dimensional stability, reduced hygroscopicity, and improved resistance to microbial decay, but are characterized by reduced mechanical properties [28].

Another approach to the esterification of lignin with carboxylic acids or their derivatives is described in the work of Thunga et al. [41], where lignin is esterified according to the procedure developed by Thielemans and Wool. Kraft lignin is reacted with butyric anhydride in the presence of 1-methylimidazole, which serves as a catalyst. After quenching the reaction with the addition of ethyl ether and separation of ether and aqueous phase, cyclohexane was added to the ether phase as a precipitating agent for the modified lignin. The precipitate was recovered by filtration and dried under vacuum for 24 h [95]. The obtained butyrated lignin was mixed with PLA using a twin-screw micro-compounder to form composite fibers. Stress–strain curves show that with an increased amount of butyrated lignin, the mechanical properties—Young modulus, strength, and strain at break—decrease significantly, owing to the amorphous structure of the lignin. The fabricated fibers were subjected to carbonization to assess their possible use as a greener alternative to commercially available polyacrylonitrile (PAN)-based carbon fibers. SEM micrographs revealed that carbonized fibers exhibit a porous microstructure, resulting from depolymerization and volatilization of PLA. The structure of carbonized fibers obtained using a 75/25 ratio of butyrated lignin/PLA is comparable with the porosity of commercial, PAN-based carbon fibers [41]. A similar procedure of obtaining butyrated lignin was applied in the work of Zhang et al. [42], where n-hexane was used instead of cyclohexane. In this work, lignin was also reacted with octadecyl isocyanate to yield lignin urethane. Both additives were incorporated into the polyurethane matrix. The results show that lignin urethane had a complex effect on the mechanical properties of the composites, increasing the Young’s modulus. Moreover, the addition of lignin urethane decreased the thermal degradation of composites at low temperatures, but at elevated temperatures, the reverse action was observed [42].

In the work by Victor, Gonçalves, and Machado [44], kraft lignin was modified with methacrylic anhydride in the presence of 1-methylimidazole. The esterification process was carried out at 50 °C under a nitrogen atmosphere for 24 h, in the presence of 1,4-dioxane, and the process of obtaining the dried and purified modified product was analogous to that described previously [31,39,41,42,95].

**Figure 4:**
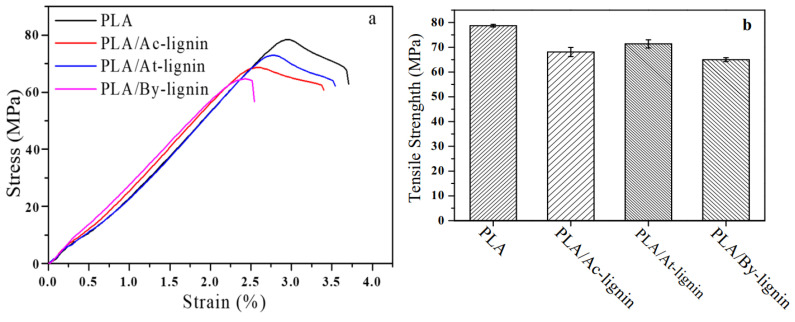
Tensile stress–strain curves (**a**) and tensile strength statistical histogram (**b**) of pure PLA and PLA/lignin biocomposites. Reproduced from [43], with the permission of MDPI.

The recently published work by Guo et al. [43] presents another route for the modification of lignin by esterification. Kraft lignin is first subjected to acidic hydrolysis in the presence of H_2_SO_4_, centrifuged, and freeze-dried. The resultant acidified lignin is dissolved in γ-valerolactone to form a homogeneous solution, and after the activation at the elevated temperature, reacted with acetyl ketene to form acetoacetate lignin, which was obtained by precipitation in ethanol, centrifugation, and freeze-drying. The modification of acidified lignin with butyric anhydride was carried out for 24 h in the presence of 1-methylimidazole. The reaction mixture was subsequently poured into water to form a precipitate, which was then centrifuged and finally freeze-dried. The obtained modified and unmodified lignins were used to produce composites with PLA matrix, where the addition of each of the fillers was 5 wt.%. PLA was blended with the additives, dried in an oven for 8 h at 80 °C, and extruded using a micro twin-screw extruder. Test specimens were formed using an injection molding process. Results of the study reveal that despite using acidified lignin as a starting block for obtaining modified lignins, each of the products presented different particle sizes and shapes. Mechanical tests show that lignin modified with acetyl ketene, which allowed for facilitated hydrogen bonding, exhibited better interfacial compatibility, and consequently, higher tensile strength and storage modulus than other composites; however, the obtained results were slightly worsened in comparison to pure PLA. Results of the thermal analysis (Figure 4) reveal that composites containing lignin modified with acetyl ketene and butyric anhydride have better thermal stability than those containing acidified lignin, though the rate of thermal degradation for all the composites was higher than that of pristine PLA [43].

Another work pertaining to the modification of lignin with carboxylic acids utilized Organosolv lignin powder which was impregnated with 5 wt.% reagent solutions of oleic acid, lactic acid, butyric acids, or butyrolactone in ethyl ether, followed by radio-frequency cold plasma modification. After the treatment, the product was removed from the reactor, unreacted reagents were extracted for 8 h in a Soxhlet extractor with ethyl ether, and the obtained modified samples were dried and analyzed. Characterization results of the modified lignin show that employing cold plasma conditions allows for successful modification of lignin with carboxylic acids, which was confirmed by XPS, FTIR, and ^1^H-NMR spectroscopies, as well as SEM imaging [49].

Sun et al. [45] described the modification of kraft lignin with succinic anhydride for obtaining a co-hardening agent for epoxy resins. Kraft lignin was dissolved in pyridine and then reacted with succinic anhydride. The product was collected by filtering the precipitate under vacuum and washed with water, followed by vacuum drying at 80 °C. The obtained modified lignin was dissolved in THF and mixed with diglicydyl ether, and after removing the solvent from the rotary evaporator, diethyl toluene diamine was added. The mixture was blended into homogeneity, poured into a glass mold, and degassed under vacuum. The resin was crosslinked at 130 °C for 1 h and 160 °C for 2 h, then post-cured at 230 °C for 4 h. The results show that the incorporation of modified lignin into the epoxy system allowed for effective reinforcement and toughening at a moderate (up to 10 wt.%) loading content. Due to the branched structure rich in phenolic content, modified lignin altered the crosslinking architecture and reduced the crosslinking density, which resulted in enhanced mechanical properties of the fabricated epoxy composites [45].

Kraft lignin modification with maleic anhydride was described by Maldhure et al. [46]. In this approach, maleic anhydride was molten by irradiation in a microwave oven, and then kraft lignin was added in small portions in a separate setup with conventional heating at 100 °C. Subsequently, the reaction mixture was remounted into a microwave reactor for 20 min with intermittently controlled irradiation. The reaction mixture was poured into cold water and filtered to recover insoluble residue (MALig). The precipitate was washed with distilled water to remove unreacted maleic anhydride and then dried for 24 h at 80 °C in an oven. The prepared maleic anhydride-modified lignin was blended with polypropylene using a mixer operating at 190 °C. Extrudates were granulated, and test specimens were obtained using the injection molding technique. In a comparative study with lignin modified with dichloroethane (CELig), blends of MALig with PP displayed better miscibility, compatibility, solubility, and better intermolecular interactions, which resulted in better overall mechanical properties. Composites containing up to 10 wt.% of MALig showed comparable mechanical properties to pristine PP, but with a higher content, a slight deterioration in mechanical properties was observed. In contrast, composites containing CELig exhibited a continuous deterioration in mechanical properties [46].

Maleic anhydride as a modifier of lignin was proposed in the work of Zhang et al. [47], who dissolved purified alkali lignin in a 20 wt.% aqueous NaOH solution, and after 1 h, it was reacted with maleic anhydride for 4 h at 70 °C. Subsequently, the mixture was precipitated using hydrochloric acid. The resultant modified lignin was vacuum-dried for 24 h at 70 °C and used to obtain polymeric composites with poly(butylene succinate) (PBS) matrix. Both PBS and modified lignin (10, 20, or 30 wt.%) were dissolved in DMF at 70 °C for 4 h and combined with 5 wt.% of one of the three different plasticizers—triethyl citrate (TEC), acetyl tributyl citrate (ATBC), or tricresyl phosphate (TCP)—then stirred for another 36 h. The homogeneous mixture was slowly cast onto an iron plate and dried in a ventilated oven for 24 h at 70 °C to evaporate the solvent. The reference sample containing 20 wt.% of unmodified lignin was also obtained analogously to the rest of the composites. SEM images of the obtained composites show that modification of lignin with maleic anhydride improved miscibility between the matrix and the filler, which was further enhanced with the addition of the plasticizers, especially in the case of TEC. The improved compatibility between modified lignin and PBS was also observable in the DSC results, where the addition of the modified lignin caused a shift of the glass transition temperature attributed to the PBS matrix towards higher temperatures, whereas the glass transition temperature connected with lignin shifted towards lower temperatures. The effect was particularly pronounced with the addition of TEC plasticizer, indicating the improved compatibility between the matrix and the filler. The introduction of both modified and unmodified lignin to the PBS matrix caused a decrease in the value of elongation at break, while tensile strength and Young’s modulus were increased. Moreover, for the composites containing 20 wt.% of modified lignin and TEC or TCP, elongation at break was improved, while both tensile strength and Young’s modulus deteriorated in comparison to composites without the plasticizers. The addition of maleic anhydride-modified lignin into the PBS matrix substantially improved the UV-blocking properties of the composites, while the addition of TCP and TEC enhanced their visible-light transmission. The authors also tested the biodegradability of the obtained composites, and while the addition of the TEC hindered the rate of microbial degradation, all the materials were completely degraded within six months [47].

In another development, alkali and de-alkali lignins were modified via transesterification with tung oil and esterification with acrylic acid or n-butyl methacrylate (BMA). Modification of lignin with tung oil was carried out for 2 h at 50–60 °C under mechanical stirring. Products were washed with acetone, transferred to a Petri dish, and dried overnight in a vacuum oven at 70 °C. For modification of lignins with acrylic acid, the reagents were placed in a flask with cyclohexane with the addition of sulfuric acid as a catalyst. The reaction was carried out for 24 h, and then cyclohexane was evaporated using a rotary evaporator. The obtained material was dried as described above. The reaction between lignin and BMA in the presence of NaOH occurred at 50 °C for 2 h. Modified lignins were mixed with a small amount of benzoyl peroxide and either BMA, styrene, or divinylbenzene (DVB) in an aluminum mold. Specimens were obtained by compression molding of the as-described mixtures at 150 °C for 1.5 h under 1500 psi pressure. The results of the study show that chemical modification of alkali and de-alkali lignins affected their properties and structure. DSC analysis of de-alkali-modified lignin showed an increase in the glass transition temperature, but in the case of modified alkali lignin, the same increase was not observed, since non-modified alkali lignin exhibits an endothermic transition at a higher temperature. TGA curves of de-alkali lignin samples show a significant decrease in moisture content after chemical modification, which can be attributed to the functionalization of hydroxyl groups with less polar moieties. Raman spectroscopy results indicate that the chemical modification of de-alkali lignin promotes partial depolymerization of the lignin network. The same effect was also observed for alkali lignin modified with tung oil. Composites obtained using modified lignins and BMA, styrene, or DVB were an attempt to create a lignin-based thermosetting resin. The authors noted that lignin samples modified with tung oil were the most versatile modifier of all the tested options and that the composite obtained by co-polymerization of acrylic acid-modified de-alkali lignin with DVB displays E’ at 25 °C and a glass transition temperature close to the Novolac epoxy resins, making it a potential greener alternative to commercially available petroleum-based resins used as the binding agents [50].

Modification of lignin via esterification using phthalic anhydride in the presence of pyridine was presented by Sailaja and Deepthi (Table 5). Esterified lignin was blended with low-density polyethylene (LDPE) and a LDPE-g-maleic anhydride compatibilizer by melt mixing at 210 °C. The use of maleic anhydride-grafted LDPE as a compatibilizer improved adhesion between LDPE and esterified lignin, which resulted in enhanced mechanical properties. Even with high loading of lignin phthalate (40 wt.%), the tensile strength values of the obtained composites were similar to that of neat LDPE. SEM micrographs of the fracture surface exhibit quasi-brittle failure for a lower content of lignin phthalate, while for a higher content, brittle fracture mode was observed. TGA of compatibilized blends showed lower weight loss, whereas an increase in lignin content contributed to facilitated char formation [48].

### 4.2. Alkylation and Arylation

**Figure 5:**
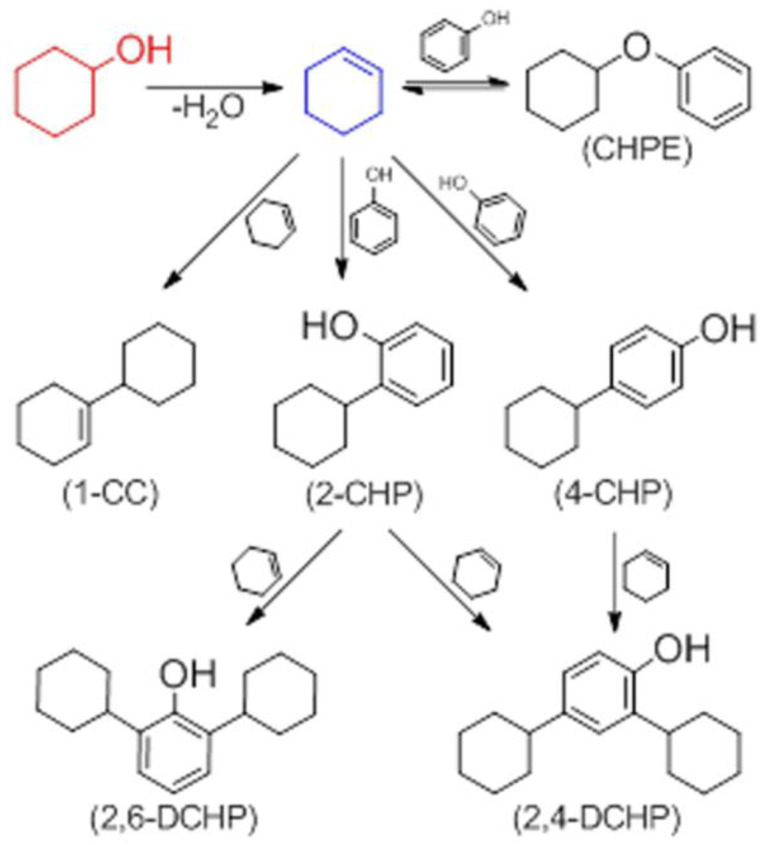
A simplified reaction network for the alkylation of phenol with cyclohexanol in decalin. Reproduced from [96], with the permission of Elsevier.

Maldhure and Ekhe [30] investigated the effect of the modification of lignin with alkyl and aryl chlorides (Figure 5) on the properties of the composites based on polypropylene. Kraft lignin was modified with dichloromethane (CMLig) and chlorobenzene (CBLig) in the presence of anhydrous aluminum chloride. CMLig was obtained by reacting lignin with dichloromethane for 1 h, then the reaction mixture was cooled, filtered, and washed with water, and the product was dried in an oven for 8 h at 80 °C. CBLig was obtained by reacting lignin with chlorobenzene at room temperature for 0.5 h, then refluxed for 2 h at boiling temperature after the chlorine gas was no longer emitted from the condenser. After cooling, the organic layer was separated. The reaction product was washed with water and dried for 6 h at 80 °C. Composites of CMLig and CBLig (5, 10, 15, 20, and 25 wt.%, respectively) with PP were prepared by melt mixing, with continuous dry nitrogen flow to minimize thermo-oxidative degradation. The obtained extrudates were granulated and testing specimens were prepared by injection molding. The results of the study show that the alkylation of lignin allowed for better compatibility of CMLig with PP, which was concluded based on better thermal stability, a more pronounced shift of the melting temperature toward lower temperatures, and better overall mechanical properties of the composites containing CMLig in comparison to those containing CBLig. It was noted, however, that the addition of the modified lignin to the PP matrix was beneficial up to 15 wt.% of the additive [30].

In another study, lignin was subjected to alkylation by butyrolactone and tetrahydrofuran to obtain structures resembling the lignin-carbohydrate complex (LCC) (Table 6). Kraft lignin was reacted with γ-butyrolactone in the presence of 1 wt.% of sulfuric acid as a catalyst for 1 h at 200 °C. After that, the temperature was raised to 250 °C and the mixture stirred for another hour. In THF modification, the reaction was also carried out in the presence of 1 wt.% of sulfuric acid, stirred for 30 min at 50 °C, followed by stirring for another 30 min at 100 °C and for 1 h at 150 °C. The presented reaction conditions were used to enable the formation of the aliphatic chains via ring-opening polymerization. The obtained modified lignin was mixed with synthetic polymers, namely polypropylene and poly(ethylene terephthalate) through melt blending using a kneader, and subsequently, compression-molded by a hydraulic press. The composites contained as high as 25 or 50 wt.% of modified lignin. Composites of PET with butyrolactone-modified lignin showed a decrease in the melting temperature and an increase in the glass transition temperature with the increase in the amount of the additive. The addition of the THF-modified lignin into the PP matrix had no significant effect on the melting temperature regardless of the amount used. The tensile strength and Young’s modulus of the PET-based composites significantly decreased with the amount of the additive, whereas the strain at break dramatically increased for 25 wt.% of the butyrolactone-modified lignin, followed by a slight increase for 50 wt.%. In the case of PP-based materials, tensile strength was decreased with the addition of THF-modified lignin, but values of both Young’s modulus and strain at break were increased [58]. A similar study by Kim et al. [59] describes the chemical modification of lignin with THF and lactic acid, which were later blended with PLA matrix using a kneader, with the loading percentages of 20, 40, and 60 wt.%, and finally, compression-molded into sheets. The results indicate that introduction of PLA-modified lignin into the PLA matrix resulted in the deterioration of mechanical properties with the increased filler content. Better mechanical properties were observed for composites containing THF-modified lignin, although the obtained values were decreased in comparison to the non-modified PLA [59].

### 4.3. Epoxidation

Chemical modification of lignin via epoxidation consisted of two steps: (i) Depolymerization of Organosolv lignin with acetone in the presence of Ru as a catalyst, carried out in the autoclave reactor purged with nitrogen. The reaction was performed at 350 °C under the pressure of 100 bar for 1 h, and after that time, the reactor was quenched with water to stop the reaction. The liquid product was dried in a vacuum rotary evaporator to remove the acetone. As-produced depolymerized lignin was then (ii) subjected to epoxidation using epichlorohydrin under alkaline conditions. The solid product was separated by filtration, washed with distilled water, and dried under vacuum for 6 h at 30 °C. The depolymerization step was essential for obtaining materials with appropriate properties for epoxy resin applications [60].

Malutan et al. [31] presented a route for chemical modification of lignin by introducing epoxy groups into its structure. Two types of alkali lignin (from wheat straw and Sarkanda grass) and hydroxymethylated Protobind lignin were used as substrates for epoxidation. As in other works, lignin was reacted with epichlorohydrin in alkaline conditions to yield two fractions of epoxidized lignin: water-soluble liquid phase and insoluble solid phase. The two phases were separated by centrifugation, washed with distilled water, and dried under vacuum conditions at 30 °C. Both hydroxymethylated and alkali lignins yielded epoxidized products, which can be used in the composite formulation for wood applications [31].

**Figure 6:**
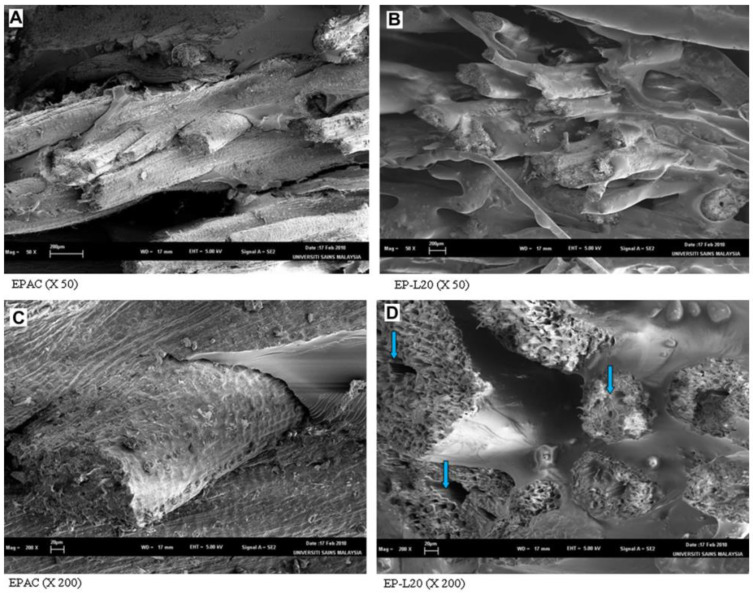
(**A**–**D**) SEM micrographs of epoxy composites at different magnification: (**A**,**C**) = Amine-cured EFB/epoxy composite—EPAC. (**B**,**D**) = Typical lignin-cured EFB/epoxy composite—EP-L20. The arrows indicate the pores on the fracture surface. Reproduced from [97], with the permission of Elsevier.

In the study by Yang et al. [61], epoxidation of lignin (Figure 6) is one of the employed methods of chemical modification for obtaining functionalized additives for polyurethane foams. Similarly, as before, alkali lignin was dissolved in the aqueous sodium hydroxide solution with a small addition of formaldehyde solution and subsequently reacted with epoxy chloropropane (Table 7). The reaction was ended by the addition of hydrochloric acid and the product was filtered out, washed with water, and dried in an oven at 80 °C. Polyurethane foams (PUFs) were obtained using a polyol mixture consisting of polyether glycol, blowing agent, catalysts, foam stabilizer, modified lignin (with content ranging from 0.5 to 3 wt.% in intervals of 0.5), and 4,4′-methylene diphenyl isocyanate. PUF prepared with the addition of epoxidized lignin exhibited a homogeneous structure, had better mechanical properties, and presented the greatest value of compressive stress and strength, as well as the most stable relative deformation, compared to pristine polyurethane foams [61].

### 4.4. Hydroxymethylation

**Figure 7:**
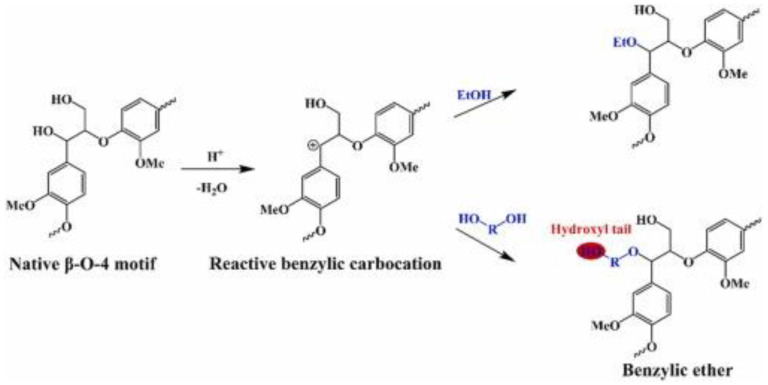
Enhancing α-etherification of lignin by diol pretreatment at a mild temperature (120 °C) to isolate lignin for producing 5 times the monomer yield of that from technical ethanol pretreatment (170 °C) by hydrogenolysis. Reproduced from [98], with the permission of Elsevier.

The incorporation of hydroxymethylated lignin into different polymeric matrices (Figure 7) resulted in:

- Achieving improved processability [63],
- Good compatibility with the matrix [63,67],
- Increased crosslinking density [63],
- Improved mechanical and thermal properties [65,67],
- Enhanced processing stability [67].

In the work by Yu et al. [53], alkali lignin was dissolved in an aqueous solution of NaOH and then a certain amount of formaldehyde was added to the mixture, which was subsequently heated to 50 °C. After the completion of the reaction, the modified lignin was precipitated by the addition of 10 wt.% solutions of HCl, and the solid residue was washed with water [53]. A similar procedure for hydroxymethylation was also employed by Yang et al. [61], where the alkali lignin modified with formaldehyde was used as an additive to polyurethane foam systems. However, the morphology analysis by SEM revealed that this type of modification is not suitable for such applications, as the miscibility of the hydroxymethylated lignin with a polyol component was poor, which resulted in filler agglomeration and a heterogeneous structure of the foam [61].

**Figure 8:**
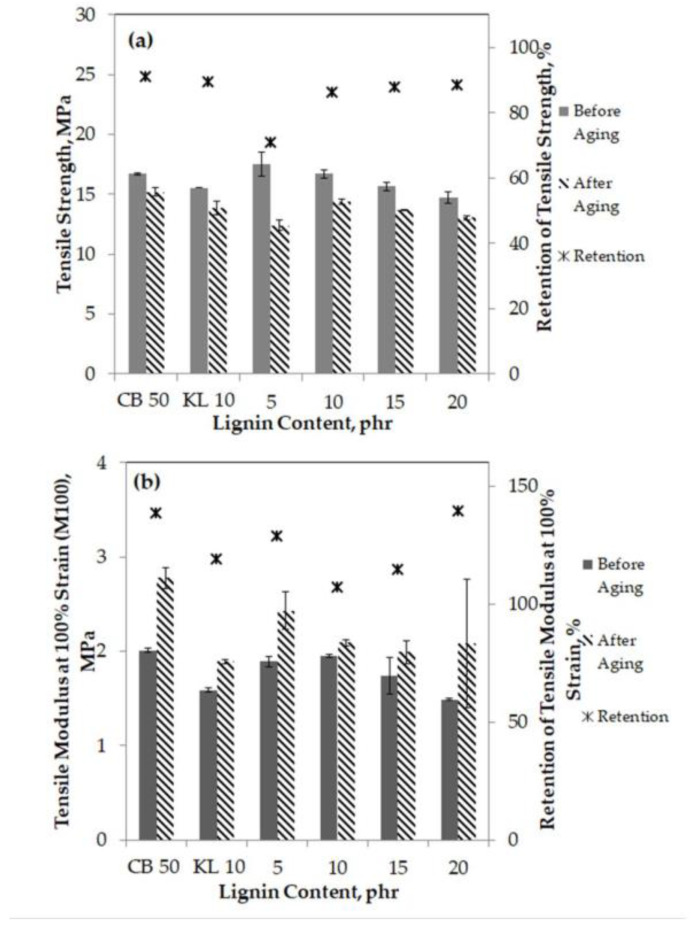
The effect of hydroxymethylated kraft lignin content on the retention of (**a**) tensile strength and (**b**) tensile modulus at 100% strain in lignin-filled NR/BR compounds. Reproduced from [63], with the permission of MDPI.

In Aini et al.’s work [63], hydroxymethylated lignin (Figure 8) was used as an additive in rubber composites. The kraft lignin was subjected to hydroxymethylation using a method proposed by Popa et al. [62], to substitute hydroxyl groups of lignin and improve the interactions between the filler and the rubber matrix. Lignin was mixed with distilled water for 2 h at room temperature. After that, 50 wt.% of NaOH solution, as well as 25 wt.% solutions of NH_4_OH as a catalyst, were added to the suspension, and the whole mixture underwent shaking for 2 h. Subsequently, the formaldehyde solution was added to the system, and the reaction was carried out for 4 h at 85 °C. The reaction product was precipitated by the addition of 1 M HCl solution, separated by centrifugation, washed with distilled water, and dried for 24 h at 50 °C. The composites of natural rubber (NR) and butadiene (BR) rubber with modified lignin were prepared using laboratory-open two-roll mills. In the first step, NR and BR were mixed with lignin, carbon black, and other additives. Then, the blend was removed and kept at room temperature for 24 h, followed by press-curing at 150 °C into sheets of 2 mm thickness. Control samples containing unmodified lignin and without the lignin were prepared analogously, as described above. Results of this study show that hydroxymethylation of lignin facilitated the formation of the methylene bridges and crosslinking. Introduction of the modified lignin into rubber composites improved the processability of the rubber and showed a good interaction between the hydroxymethylated lignin and the matrix, increased the crosslinking density, and as a result, increased the curing rate of the rubber compounds in comparison to composites with unmodified lignin. The mechanical properties of rubber composites containing modified lignin were improved up to 10 parts per hundred rubber (phr), but the aging resistance and thermal stability did not differ from the values obtained for composites filled with unmodified lignin [63]. A similar approach for lignin modification by hydroxymethylation was presented in the work of Gilca et al. [64], where the reaction between alkali lignin and formaldehyde was applied to obtain chemically modified lignin nanoparticles [64].

Hydroxymethylation of lignin was also performed in the study by Xiao et al. [65], where kraft lignin was subjected to a reaction with formaldehyde under alkaline conditions at 90 °C for 3 h (Table 8). The solid product was obtained by a series of purification processes of acidification and ultra-filtration, followed by spray-drying. In order to obtain composites of poly(propylene carbonate) (PPC) with hydroxymethylated lignin, the polymer and the filler were compounded in a micro twin-screw extruder at 170 °C, with the modified lignin content of 10/100, 20/100, 30/100, and 40/100. The extrudate was granulated and shaped into specimens on a flat vulcanizing machine at 180 °C under 10 MPa for 1 min. The result of the mechanical testing showed that both the tensile strength and the tensile modulus of the composites were higher than that of neat PPC; however, the mechanical properties of the composites deteriorated with the increasing amount of hydroxymethylated lignin, probably due to the filler aggregation. Moreover, all the composites had improved thermal and processing stability in comparison to pristine PPC [65].

Along this line of interest, Jiang et al. [67] studied how the addition of hydroxypropylated lignin will affect the properties of poly(propylene carbonate) (PPC) composites. Kraft lignin was dissolved in deionized water and reacted with propylene oxide under alkaline conditions for 10 h at 30 °C. The solid hydroxypropylated lignin was obtained after purification, followed by spray-drying. The so-prepared modified lignin was mixed mechanically with PPC at weight ratios of 10/100, 20/100, 30/100, and 40/100, and extruded using a micro twin-screw extruder. The obtained composites were granulated and shaped into sheets on a flat vulcanizing machine at 180 °C under 10 MPa pressure. As a control specimen, neat PPC and PPC with unmodified lignin sheets were also processed according to the described procedure. Incorporation of the polypropylene chains into the structure of lignin improved its compatibility with the PPC matrix, which can be observed on the SEM micrographs, where in the case of composites containing unmodified lignin, there is a clear phase interface, whereas for composites with the addition of hydroxypropylated lignin, the phase interface between the matrix and filler was blurry. Moreover, the tensile strength, tensile modulus, and thermal stability of the composites were significantly improved with the addition of the modified lignin in comparison to the neat matrix. Hydroxypropylation of lignin also positively influenced the processing stability of the composites and their ability to undergo microbial degradation [67].

Another interesting pathway is oxypropylation of lignin to yield polyol-like additives for rigid polyurethane foams. Different lignin samples (kraft, soda, and Organosolv) were reacted with propylene oxide in the presence of KOH as a catalyst. The reaction products containing both oxypropylated lignin and propylene oxide oligomers were not separated, as both are reactive toward the components of the polyurethane systems. Interestingly, the obtained lignin-based polyols were characterized by hydroxyl number and viscosity values close to commercially available polyols used in rigid polyurethane foams’ production [32].

### 4.5. Copolymerization

**Figure 9:**
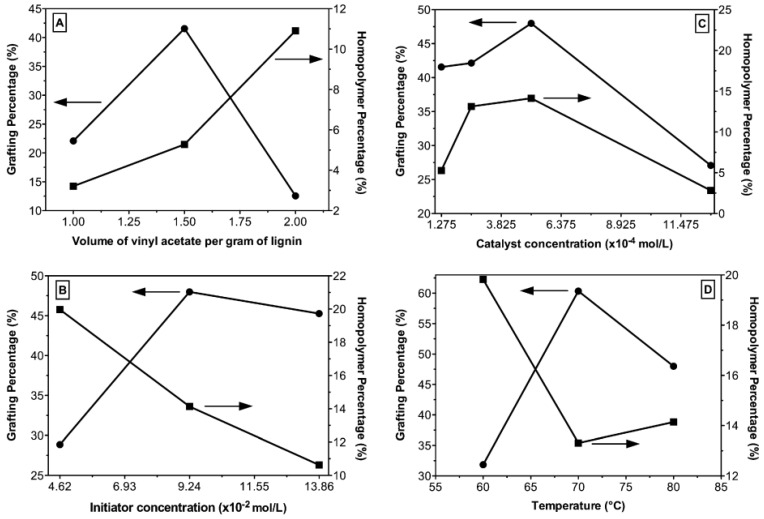
Effect of reaction conditions on graft copolymerization. Reproduced from [100], with the permission of Elsevier. (**A**) The effect of monomer concentration on grafting percentage and homopolymer formation, (**B**) The amount of potassium persulfate used in the reaction medium in the graft copolymer formation, (**C**) The effect of catalyst concentration on grafting percentage and homopolymer formation, (**D**) The temperature influence on grafting percentage and homopolymer formation.

Introduction of various side chains in the lignin structure via copolymerization (Figure 9) resulted in enhanced metal ion absorption properties [68,99], improved mechanical properties [69,70,71], free radical inhibition [69], and better UV-blocking properties [71].

Copolymerization as a method of introducing new structural elements into the polymeric architecture was proposed in numerous works, such as in the study by Zhou et al. [68], who modified lignosulfonate by N-methylaniline. To obtain a functionalized additive, lignosulfonate was dissolved in an ammonia solution, and then N-methylaniline was added to the mixture and stirred. Then, ammonium persulfate solution was added to the mixture to initiate polymerization, which occurred at 25 °C for 24 h. Finally, the reaction mixture was filtered and washed with ammonia solution and deionized water, followed by drying of the product at 60 °C for 24 h. The as-obtained composites were tested for Cr(VI) removal from the aqueous solutions, and the results show a good maximum adsorption capacity of the composites. Moreover, the lignosulfonate/N-methylaniline copolymers showed good selectivity of Cr(VI), which is promising in the application of such materials as alternative, bio-based adsorbents for wastewater purification [68].

The work of Kai et al. [69] highlights the copolymerization of lignin with esters to form sustainable nanofibers with antioxidant properties with potential in healthcare applications (Table 9). Alkali lignin was reacted with ε-caprolactone and L-lactide with the addition of tin(II) 2-ethyl hexanoate as a catalyst in solvent-free conditions, to introduce side chains of poly(ε-caprolactone-co-lactide) (PCLLA) via ring-opening polymerization. To obtain nanofibers by electrospinning, mixtures of polycaprolactone (PCL) and obtained lignin-PCLLA copolymers were dissolved in 1,1,1,3,3,3,-hexafluoro-2-propanol (HFP) to yield 10 wt.% solutions. After one day of stirring, the homogeneous solution was placed in the syringe and pumped out at 0.8 mL/h with a voltage of 12 kV applied to the needle. The fibers were electrospun onto an aluminum foil-wrapped collector and after the spinning, obtained fibers were dried overnight in the oven under vacuum conditions. Composite nanofibers of poly(L-lactic acid) (PLLA) and PCLLA-modified lignin were obtained analogously to those described above, with the exception of the concentration of the spinning solution, which was 8 wt.%. The obtained nanofibers were characterized by uniform, beadless, randomly oriented structures, which indicates uniform dispersion of modified lignin particles in polyester matrices. Moreover, the addition of lignin-based composites as an additive to PCL and PLLA matrices increased their rate of degradation. The study results also show that obtained electrospun nanofibers are biocompatible and non-cytotoxic, which indicates their potential for biomedical or healthcare applications [69].

Nair and co-workers copolymerized lignin with chitosan to obtain materials for potential use in dyes or metal ions’ absorption from wastewater. Alkali lignin was dispersed in distilled water and stirred, while chitosan was dissolved in the 2 wt.% aqueous acetic acid solution to form a homogenous solution. The dispersion of alkali lignin was added to the chitosan solution and stirred for 3 h. After that, the mixture was filtered, and the solid residue was dried at room temperature for 48 h. The obtained material was powdered, double-washed with distilled water, and vacuum filtered, then finally dried at 100 °C for 3 h. According to the procedure described above, copolymers of chitosan containing 5, 10, 25, and 50 wt.% of lignin were obtained. Since the evaluation of the adsorption properties was the highlight of the work, the results indicate that the copolymer containing 50 wt.% of both chitosan and lignin displayed maximum adsorption of Remazol Brilliant Blue R dye and Cr(VI) in comparison to other copolymers and pure chitosan. The adsorption efficiency was also confirmed for the range of other dyes belonging to anthraquinone, mono-azoic, and triphenylmethane families. Obtained results show potential in the application of such biopolymer materials as low-cost adsorbents for wastewater purification processes [99].

In another approach, lignin was modified by the introduction of side chains via ring-opening polymerization of L-lactide. Lignin and L-lactide in a 10/90 ratio were weighed into a sealed reaction vial and the reaction in the presence of triazabicyclodecene as a catalyst was carried out for 3.5 h at 130 °C. Subsequently, the reaction mixture was cooled and quenched with a dichloromethane solution of acetic acid. After the dissolution of the residue, the reaction mixture was evaporated on a rotary evaporator to obtain a highly viscous solution, which was then poured into a methanol solution to precipitate. The precipitated polymer was collected by extraction with dichloromethane. The polymers were concentrated and dried under vacuum conditions to a constant weight. Copolymers containing 30–50 wt.% of lignin were synthesized similarly to those described above but were purified by dialysis over methanol, followed by extraction with dichloromethane and concentration under vacuum conditions. The as-prepared copolymers as well as non-modified lignin were blended with PLA in chloroform at room temperature for 15 h. The mixture was then precipitated using isopropanol and the solid residue was dried in a vacuum oven for 3 days at 80 °C. The composite specimens were obtained by hot pressing the dried precipitate at 190 °C and cut into samples for testing. Results of the reported study show that the addition of the lignin-PLA composite into the PLA matrix allows to induce UV-blocking properties. The incorporation of copolymers resulted in an increased tensile strength and strain without deterioration of the tensile modulus [71].

Lignin/poly(3-hydroxybutyrate) composite nanofibers were prepared by Kai et al. [70] using the electrospinning technique. Alkali lignin was modified by the introduction of side chains by ring-opening polymerization of β-butyrolactone and ε-caprolactone. There were four pathways of modification introduced in this study. The first consisted of a reaction of the lignin with β-butyrolactone in the presence of tin(II) 2-ethyl hexanoate as a catalyst, performed for 8 h at 130 °C. After that, the mixture was cooled and the copolymer was dissolved by the addition of chloroform, then the mixture was separated by centrifugation. The supernatant containing dissolved copolymers was precipitated twice in ether. The obtained lignin-PHB copolymer was dried overnight in an oven at 50 °C under vacuum conditions. To obtain a random lignin-PHB-PCL copolymer, lignin was reacted with equal amounts of β-butyrolactone and ε-caprolactone for 8 h with the catalyst, followed by precipitation and drying. To obtain block copolymers of lignin, lignin was reacted with either β-butyrolactone or ε-caprolactone for the first 4 h, and after removal of the unreacted monomers by precipitation, the reaction was continued for another 4 h with the addition of the other monomer, followed by precipitation and drying. The obtained copolymers were blended with PHB and dissolved in 1,1,1,3,3,3,-hexafluoro-2-propanol. After the homogeneous blend was obtained by stirring overnight, the solution was placed into a syringe and pumped out at a rate of 1 mL/h with 10 kV of the voltage applied to the needle. The nanofibers were collected on the collector wrapped in aluminum foil, and after the electrospinning, the obtained nanofibers were dried overnight under vacuum conditions. The addition of the lignin-PHB copolymer into PHB decreased the tensile strength and Young’s modulus of the fibers, while the elongation at break increased. In the case of random lignin-PHB-PCL and block lignin-PHB-PCL copolymers, all three mechanical parameters decreased. The best results for mechanical testing were obtained for the lignin-PCL-PHB copolymer, where all three parameters had enhanced values. In the study of biodegradation, nanofibers containing lignin-based copolymers showed better stability to degradation caused by soaking in phosphate-buffered saline, and biocompatibility test results revealed non-cytotoxicity of the nanofibers [70]. In another work by Kai and co-workers, an analogous method of obtaining lignin-PHB copolymers was employed, and once again, the electrospun composite nanofibers of PHB and modified lignin were prepared. The addition of 2% of lignin-PHB copolymer resulted in an increase of tensile strength, stiffness, and elongation at break in comparison to pure PHB. The nanofibers were characterized by antioxidant activity and biocompatibility, which can indicate potential application in biomedicine [72].

### 4.6. Amination

Utilizing amination as a method of lignin modification results in:

- Imparting hydrophilicity [73],
- Better adsorption capacity of metal ions [74,75],
- Better thermal stability [77,79,80],
- Enhanced mechanical properties [77,78],
- Anti-aging properties of polymeric composites [78].

**Figure 10:**
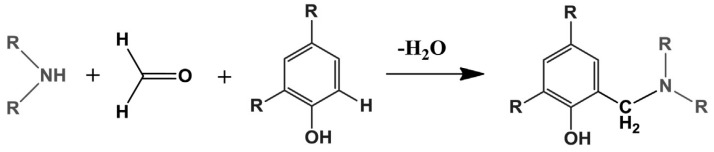
Mannich reaction mechanism utilized in the amination of lignins.

The amination of lignin with various amine compounds in the presence of formaldehyde occurs via the mechanism of the Mannich reaction (Figure 10), and numerous works have addressed this type of chemical modification. In their work, Du et al. [73] compared the modification of lignin using the Mannich reaction with and without phenolation pretreatment. As starting materials, LignoBoost lignin (LBL) and 4-hydroxy-3-methoxyacetophenone (HMAP) as a lignin model were used. For phenolation, LBL and phenol were mixed with 72 wt.% of sulfuric acid and stirred for 6 h at 60 °C, then the solution was diluted with water to reach 3.0 wt.% of sulfuric acid. The resultant solution was autoclaved for 1 h at 125 °C. The solid residues (P-LBL) were filtered, washed with deionized water, and dried in an oven at 105 °C. The Mannich reaction was conducted for HMAP, LBL, and P-LBL. According to the procedure described by Matsushita and Yasuda [101], HMAP was dissolved in dioxane, followed by the addition of 40 wt.% of dimethylamine aqueous solution, 37% formaldehyde aqueous solution, and acetic acid. Then, the mixture was heated to 60 °C and stirred for 4 h. The reaction was terminated by pH adjustment to 9 with 2 M NaOH, followed by extraction with ethyl acetate. The obtained solution was dehydrated over sodium sulfate and evaporated to dryness by film evaporation. Similarly, for LBL and P-LBL samples, lignin was dissolved in 80% dioxane aqueous solution, followed by the addition of 40 wt.% of dimethylamine aqueous solution, 37% formaldehyde aqueous solution, and acetic acid. The mixture was heated to 60 °C and stirred for 4 h. After that, the mixture was evaporated under a vacuum to remove unreacted organic reagents, purified by dialysis, and freeze-dried. The results show that under acidic conditions and using secondary amines, the Mannich reaction occurs selectively and completely at the C-5 position on the guaiacyl aromatic rings for both the model lignin structure and LignoBoost lignin, and the preceding phenolation treatment largely increases the phenolic aromatic rings’ content, which allows for a higher degree of amination. Aminated lignin products, regardless of phenolic pretreatment or a lack thereof, display high hydrophilicity, dispersion, and protonation, as well as a positive charge under acidic conditions, which indicates their potential for various practical applications [73].

In another development, alkaline lignin dispersed in water was mixed at 50 °C with NaOH to introduce alkaline conditions. Subsequently, methylamine was added to the mixture, and the temperature was elevated to 90 °C. Formaldehyde was added dropwise for 2–6 h to enable the Mannich reaction to occur. After that, the 0.1 M HCl was used to precipitate the product, which was then filtrated, washed with distilled water, and dried overnight under vacuum conditions at 65 °C, followed by grinding. As-prepared amine-functionalized lignin was tested for the removal of lead ions from aqueous solutions by adsorption. The results show the good adsorption capacity of aminated lignin, which displays the potential use of such material as a bio-based, biodegradable biosorbent for wastewater treatment [74]. A slightly modified approach was used in the work of Qin et al. [75], where lignin was dissolved in water under alkaline conditions, then the solution was transferred into the three-necked flask with poly(ethylene imine) and the temperature was raised to 90 °C. After that, formaldehyde was added dropwise and the Mannich reaction was carried out for 5 h while stirring. After the completion of the reaction, the mixture was cooled to 4 °C and carbon disulfide was carefully added for esterification under stirring for 2 h. Finally, the mixture was filtered and washed with ethanol and distilled water. A poly(ethylene imine)-anchored lignin composite (PLCD) was obtained after drying the resultant solids under vacuum conditions at 50 °C for 24 h. The obtained poly(ethylene imine)/lignin composite containing dithiocarbamate groups exhibited a porous structure suitable for the absorption of heavy metals from water. The adsorption of Cu(II), Zn(II), and NI(II) ions was pH-dependent and most efficient at a pH of 6. PLCD was also tested for reusability by regeneration with HCl and showed good performance after five cycles. The obtained composite displayed characteristics of a proper heavy-metal adsorbent, which indicates potential uses as an alternative for commercially available sorbents [75].

In a modified version of the lignin amination method proposed by Yue et al. [102], kraft and alkali lignin were modified with DETA according to the following procedure: Lignin and DETA were dissolved in distilled water, then the pH of the solution was adjusted to 10–10.5 with the addition of 0.1 M NaOH and HCl solutions. The mixture was poured into a three-necked flask and formaldehyde solution was added at room temperature while stirring. After heating the solution at 50 °C for 4 h, isopropyl alcohol was added to precipitate the obtained aminated lignin. The residue was recovered by suction filtration, washed with isopropyl alcohol three times, and dried in the oven at 40 °C under vacuum conditions. The obtained aminated lignin was used as a modifier in the waterborne polyurethane (WPU) formulations. The formulations were obtained by adding poly(propylene glycol) and toluene diisocyanate into a four-necked flask and stirring under the nitrogen atmosphere for 2 h at 65 °C. Subsequently, the dimethyol propionic acid dissolved in acetone was added dropwise and the reaction was performed at 80 °C until the -NCO content reached the desired value. After cooling the mixture to 40–50 °C, acetone was added to lower the viscosity, followed by the neutralization of carboxylic acid groups by the addition of triethylamine. Then, the emulsification was carried out by the addition of an iced lignin amine water solution under vigorous stirring to produce WPU latex with 20 wt.% of solid content. After the removal of acetone, the latex was cast onto a Teflon mold and cured at room temperature, which resulted in obtaining WPU films of 0.6 mm thickness, which were finally dried in a vacuum oven at 50 °C for 10 h. Results of the tests performed for the obtained WPU films indicate that the mechanical properties of the WPU containing aminated lignin were significantly improved. Moreover, since pure lignin exhibits radical scavenging properties, WPU-containing modified lignin showed excellent anti-aging performance [78].

Modification of the lignin via the Mannich reaction was also employed in the work by Zhang et al. [42], where alkaline lignin was reacted with urea according to the method presented by Matsushita et al. [103] to be used as an additive in polylactide (PLA) composites. Alkali lignin was dissolved in 0.1 M NaOH aqueous solution, and the urea and formaldehyde were added. The mixture was refluxed at 70 °C for 10 h with constant mechanical stirring. After the reaction, the pH of the viscous product was adjusted to 3 using 1 M hydrochloric acid and centrifuged to obtain the solid urea-modified lignin (UM-Lig) (Table 10). The product was washed several times with water and dried under vacuum conditions at 60 °C. PLA, alkaline lignin, UM-Lig, and ammonium polyphosphate (APP) were dried overnight in the oven at 80 °C. All composite samples were prepared on a two-roll mixing mill at 180 °C, and roll speed was maintained at 40 rpm. Firstly, PLA was added into the mill, and after melting, APP and lignin or UM-Lig were added into the mix with varying ratios, maintaining 23 wt.% of total additive content in the composite. The molten mixtures were processed for 10 min until a good dispersion was achieved. The resulting samples were compressed and molded into sheets. The results from this study show that the introduction of both UM-Lig and APP enhances the flame retardancy of PLA, with the optimal ratio of APP:UM-Lig being 4:1, which allows for reaching the LOI value of 34.5 and the V-0 rating in the UL-94 test. Cone calorimeter results show that PHRR and THR values obtained for PLA/APP/UM-Lig composites are significantly reduced in comparison with PLA/APP and PLA/APP/lignin systems. TG curves indicate that the introduction of APP/UM-Lig enhances the thermal stability of PLA and promotes the formation of char. Nevertheless, the introduction of flame retardants into PLA causes deterioration of composites’ mechanical properties in relation to pure matrix [80].

Glycination of kraft lignin to obtain modifiers for hyaluronan hydrogels was proposed in the work of Musilová et al. [81]. Kraft lignin dried for 6 h at 60 °C was modified with glycine by the Mannich reaction, as described in the work by Brežný et al. [33]. Kraft lignin was mixed with glycine and sodium acetate trihydrate solution in acetic acid, and subsequently, formaldehyde was added to the solution at 50 °C. Glycinated lignin was dissolved in water for 4 h, then pH was adjusted to 2, followed by coagulation by ethyl acetate. The residues were vacuum-dried at 60 °C. Hyaluronan hydrogels without modifiers were prepared according to the previous work of Minařík’s team [104]. Hydrogels containing glycinated lignin were prepared according to the following procedure: 1% (*w*/*w*) kraft lignin solution in water was prepared by the slow addition of the appropriate amount of the polymer to Milli-Q water with continuous stirring, followed by dissolving at 50 °C for 24 h. The prepared solution was filtrated through 0.45 µm pore-size PTFE syringe filters and then mixed with the previously prepared 1% (*w*/*w*) hyaluronan solution and stirred at 50 °C for 1 h. Then, N-(3-dimethylaminopropyl)-N’-ethylcarbodiimide hydrochloride (EDC), acting as a crosslinker, was added into the solution, followed by stirring at 25 °C for 1 h. Subsequently, 0.1 M HCl solution was added to adjust the pH to 4.5–4.7 and the resultant solution was poured into a polystyrene bin and frozen for 60 h at −18 °C, followed by freeze-drying to yield a porous hydrogel in the form of a cylinder. Analyses performed for the obtained samples show that lignin was an active agent in a process of crosslinking the hydrogels with EDC. The introduction of more than 5 wt.% of lignin resulted in an increase in the pore dimension of hydrogels and lyophilized fragility. The water uptake capacity equilibrium of hydrogels decreased markedly with the addition of glycinated lignin. Moreover, the addition of lignin up to 3 wt.% enhanced the mechanical properties of hydrogels, namely the creep resistance and creep recovery were improved, whereas for hydrogels containing more than 5 wt.% of the modifier, an increase in elasticity was observed. The authors point out that the addition of glycinated lignin into the hyaluronan hydrogels does not negatively influence the cytotoxicity of the product, thus making it suitable for tissue engineering and other biomedical applications [81].

### 4.7. Silylation

Utilization of silicon compounds in the chemical modification of lignin results in:

- Enhanced thermal stability [82,83,86,87],
- Better mechanical properties [40,85,87],
- Decreased volume shrinkage [40],
- Hydrophobicity [86],
- Better compatibility with polymer matrix [86],
- Flame-retardant properties [87].

In the silylation process, silicon-containing moieties are introduced into the structure of lignin. The work by Bula, Klapiszewski, and Jesionowski on the fabrication of functional silica/lignin hybrid material and its potential use as a bio-based filler for polypropylene (PP) has been described. Kraft lignin and silica were combined mechanically via grinding and mixing using a planetary ball mill for 12 h with intervals of 15 min after the direction of rotation was changed. The whole process took 12 h, and to avoid overheating of the material, every 2 h the mill was switched off for 5 min. After the milling, the obtained hybrid material was sifted through a sieve with a mesh diameter of 40 µm, and the final product was subjected to testing. Composite materials of PP and silica-modified lignin were prepared in several steps to avoid the separation of powdered filler and matrix pellets. PP pellets were fed into the pulverizing mill and the resultant polymer flakes were mixed with other components, and thus-prepared batches were dried in an oven at 80 °C for 12 h. Composites containing 1 wt.% of PP grafted with maleic anhydride copolymer as a compatibilizer and 2.5, 5.0, or 7.5 wt.% of lignin filler (both modified and unmodified) were melt-blended in a corotating twin-screw extruder at a barrel temperature range of 190–205 °C and screw rotation speed of 150 rpm. Extrudates were then granulated, and injection-molded into testing specimens. Analyses performed for the obtained filler confirm the formation of the hybrid silica/lignin material. Moreover, TGA confirmed the enhanced thermal stability of the product in relation to raw lignin material. As for the TG curves obtained for composites, the addition of the hybrid filler did not alter the mechanism of thermal degradation of PP, whereby mechanical testing of the composites shows that composites containing unmodified lignin displayed a higher value of elongation at break than pure PP, and for composites with hybrid particles, the same parameter was enhanced in the case of 2.5 wt.% of silica-modified lignin [82].

**Figure 11:**
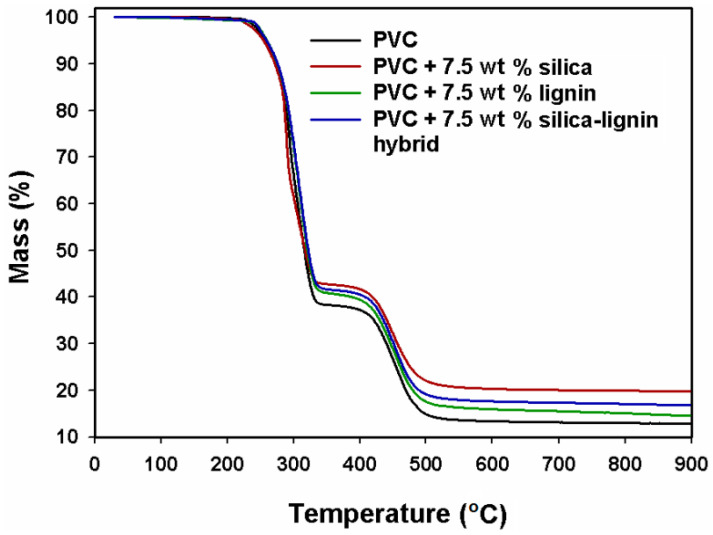
TG curves of PVC and PVC-based composites with a 7.5 wt.% filler content. Reproduced from [83], with the permission of MDPI.

Results of this study were employed in the work of Klapiszewski and co-workers, where fillers prepared as described above were incorporated into the PVC matrix at 0, 2.5, 5, 7.5, and 10 wt.% concentrations. PVC composites were processed by melt kneading in a Brabender mixer at 190 °C and 30 rpm. Meanwhile, for composites containing 10 wt.% of the filler, the degradation process was observed during the mixing, so further examination of the material was discarded. After the processing, all the obtained materials were ground and compression-molded into specimens at 180 °C and cut into testing samples. The results show (Figure 11) that the incorporation of the hybrid filler into the PVC matrix resulted in a uniform composite structure, enhanced values of Young’s modulus in comparison with pristine PVC, and a slight decrease in tensile strength. Composites of PVC and hybrid silica-lignin filler also displayed an elevated Vicat softening temperature as well as better thermal stability [83]. Another study of this group, by Borysiak, Klapiszewski, Bula, and Jesionowski, concentrates on how silica/lignin filler in composites with polypropylene affects their nucleation ability. Kraft lignin was combined with silica in a mechanical process, where the powders were ground and mixed using a grinder mortar for 2 h, with a 2 min break after each 30 min of grinding to avoid overheating. As in the previous work, after grinding, hybrid lignin/silica fillers were sifted on the 40 µm sieve. PP pellets were mixed with hybrid fillers and lignin using a tumbler agitator, then dried in an oven for 12 h at 80 °C. Dried components were processed by melt blending in a twin-screw extruder at a 160–200 °C barrel temperature and 150 rpm. Extrudates were then injection-molded into dumbbell-shaped specimens. The results of the concluded tests indicate that the fillers with increased silica content allow for a high nucleation ability of the composites and enhanced thermal stability. Moreover, increased silica content affected the structural properties of the materials, causing the occurrence of smaller particles, a larger specific surface area, and a larger pore volume, as well as a smaller pore surface [84].

Buono et al. [86] employed modification of lignin with silanes and compared properties of the products with those obtained by acetylation. Alkaline soda lignin was subjected to both acetylation and silylation. For the first modification, 2 g of lignin was dissolved in 8 mL of pyridine, which was followed by the addition of acetic anhydride, and the mixture was stirred for 18 h at room temperature. Partially acetylated products were obtained by the addition of ethanol and then removed under vacuum, whereas fully acetylated products were precipitated by the addition of 1% HCl at 0 °C. The precipitate was filtered and washed with deionized water, then dried overnight at 40 °C in the vacuum oven to obtain acetylated lignin. For the silylated lignin, 1 g of alkali lignin was mixed with 5 mL of DMF, and the mixture was stirred until the lignin was dissolved. The silylating agent, tert-butyldimethylsilyl chloride (TDMSCl), and imidazole were added, and the reaction was carried out at room temperature for up to 18 h. In the case of partially silylated products, after the evacuation of DMF under vacuum, the product was washed with methanol. Fully silylated products were obtained by precipitation occurring after the addition of deionized water, and the precipitate was filtered and washed with methanol. Silylated lignins were dried in the oven at 40 °C under vacuum conditions. Composites of LDPE with 10 wt.% of lignin in the form of strands were obtained by extrusion in a twin-screw compounder at 110 °C and 100 rpm, then cut into 5 mm pieces and injection-molded into dumbbell specimens at 130 °C, whereas the molding temperature was set at 65 °C. The results of the study show that modification of lignin via silylation yields products with better thermal stability, more hydrophobic, and soluble in a wider range of organic solvents than the acetylated lignin (Table 11). Characterization of LDPE-lignin composites shows that silylated filler displayed enhanced compatibility with the matrix compared to acetylated lignin, which was confirmed by SEM imaging and thermal analysis data [86].

Lignin modified with γ-divinyl-3-aminopropyltriethoxysilane as an additive for poly(lactic acid) was described in the work by Song et al. [87], where 0.5 g of the silane agent, γ-divinyl-3-aminopropyltriethoxysilane (DVAPTS), was dissolved in 50 mL of a 5:95 (*v*/*v*) mixture of water and ethanol. The solution was transferred into 10 g of dried wheat straw soda lignin and stirred. The product (CLig) was then dried at 105 °C under an electric heat oven for 12 h. PLA and the flame-retardant ammonium polyphosphate (APP) were dried in a vacuum oven at 80 °C for 48 h. Composites of PLA containing 23 wt.% of additives with different ratios of APP and CLig or pristine lignin were prepared using a mixer at 180 °C and 30 rpm for 10 min, and hot-pressed under 10 MPa for 5 min at 180 °C for preparation of the testing specimens. The results of this study indicate that modification of lignin with DVAPTS resulted in better thermal stability in comparison to the unmodified lignin. As for the flame-retardant effect, the best results were obtained for the composite containing APP and CLig with a 4:1 mass ratio, which passed the UL-94 V-0 rating and obtained a LOI value of 30.5%. PHRR of PLA/APP/CLig was reduced in comparison to PLA/APP/Lig composites and had a bigger mass of charred residues. Results of the thermogravimetric analysis point to the formation of intumescent char on the surface of the composite at lower temperatures, which leads to overall better thermal stability. Moreover, PLA/APP/Clig displayed good mechanical properties despite the incorporation of the flame retardant [87].

### 4.8. Other Approaches to Chemical Modification of Lignin

Komisarz et al. [2] in their work presented a different approach to lignin modification. The sulfonyl groups present in the aromatic network of lignosulfonates were subjected to a two-step modification procedure to yield a sulfonamide derivative of lignin: first, to obtain lignosulfonyl chloride by means of a reaction of lignosulfonate with chlorosulfonic acid, and second, to obtain lignin sulfonamide by a reaction with a secondary amine. FTIR results confirmed the presence of the alkyl chains and the formation of the bond between sulfur and nitrogen in the sulfonyl group. The thermal stability of the modified lignin was considerably improved in relation to the raw materials, especially when looking at changes in the T5% temperature—the final modified material showed an improvement in the said parameter of 115 °C, in comparison with the raw material. Moreover, lower amounts of char residue were present at 600 °C, which may be caused by the change in the thermal degradation mechanism owing to the introduction of the nitrogen-based compounds into the structure of the investigated lignin derivatives. The authors suggested that the formation of the sulfonamide may retard the desulfonation, which in turn enables the improvement of the thermal-degradation parameters [2].

In their further work [3], sodium lignosulfonate was subjected to a two-step modification procedure. The first stage employed different chlorinating agents—SOCl_2_ (Route A) and PCl_5_ (Routes B and C) —to obtain lignosulfonyl chlorides, which were reacted in the second stage with dihexylamine to yield sulfonamide derivatives of lignin. Results of the FTIR spectroscopy indicate the existence of alkyl chains in the modified materials and confirm the bonding between sulfur and nitrogen in the sulfonamide group. After the first step of modification, for the lignosulfonates modified with SOCl_2_ (Route A), the thermal degradation started at lower temperatures, propagated at a slower rate, and reached the maximum mass loss at higher temperatures. Samples modified with PCl_5_ (Routes B and C) displayed thermal decomposition similar to the raw material, reaching the maximum mass loss below 200 °C. The T5% temperature of the obtained lignosulfonyl chlorides was enhanced in comparison with the raw materials. After the second step of the modification procedure, materials obtained via the reaction with dihexylamine exhibited an increase in the value of T5% and T20% temperatures. The described two-step method of the chemical modification of lignosulfonates, involving both chlorination and the formation of lignin sulfonamide derivatives by the reaction with secondary amines, may potentially be used to obtain functional, value-added lignin-based materials, which can be used as a filler in biopolymer matrices.

Sorption characteristics of acid hydrolysis lignins [105], commercial by-products of wood conversion to fuel ethanol, and their nitrogen-containing derivatives have been examined to determine the most suitable areas of application of lignin-based sorbents. Lignocellulosic residues, by-products from the conversion to ethanol, have been characterized as a raw material for sorbents. It has been established that these residues exhibit high sorption activity towards phenols and nitrogen-containing aromatic compounds. The obtained results have shown that the sorption capacity for organic contaminants of an aromatic nature increases significantly as a result of the modification of hydrolysis lignin with quaternary ammonium compounds. The amination of lignin with epoxy amines enhanced its sorption activity towards heavy metals.

In other work [106], a lignocellulosic residue was obtained in a pilot plant by dilute acid hydrolysis of coniferous wood. Two lignin residues were obtained by the concentrated hydrochloric acid process. These samples were washed with warm water to pH 5.5, and then with sodium nitrate to remove any inorganic chlorine, followed by washing with water and drying.

Liu et al. [91] modified alkali lignin with polyethyleneimine (PEI) by mixing 25 g of lignin, 6 g of formaldehyde, and 6 g of PEI and dissolving all reagents in 200 mL of distilled water, followed by the adjustment of the pH to 10 with 20 wt.% solutions of NaOH. The reaction was carried out at 50 °C for 5 h and then pH was adjusted again, this time to 3–4 using 10% HCl. The reaction product (A-lignin) was obtained by precipitation using distilled water, washed until neutral pH, and dried at 80 °C under reduced pressure until constant weight. Then, 5 g of A-lignin, 0.081 mL of formaldehyde, and 1.38 g of diethyl phosphite (DEP) were dissolved in 50 mL of DMF and a small amount of HCl solution was added to adjust the pH to about 5. The reaction was conducted at 70 °C for 5 h, then 5 g of copper acetate was introduced into the solution and the reaction continued at the same temperature for another 5 h. The product was obtained by evaporating DMF using the rotary evaporator and washed with distilled water a few times. The obtained solid material (F-lignin) was dried under reduced pressure at 80 °C until constant weight. Such modified material was used in composites of wood powder (WP) and polypropylene (PP), manufactured by melt compounding in a ThermoHaake Torque Rheometer at 180 °C for 10 min with a 60 rpm rotor speed. The incorporation of F-lignin into the PP/WP composites resulted in enhanced thermal stability and flame retardancy in comparison to composites containing unmodified lignin and without lignin. A 30% reduction in smoke formation was also observed as well as the formation of a continuous char layer, which is responsible for better flame retardancy [91]. Such an approach was also incorporated in another work by Liu’s team, in which alkali lignin was once again functionalized with PEI according to the aforementioned procedure, but the following step was altered. Similarly, as before, 5 g of A-lignin, 0.081 mL of formaldehyde, and 2.07 g of DEP were dissolved in deionized water while stirring, and subsequently, 2 wt.% of NaOH aqueous solution was added to adjust the pH of the mixture to about 10. The reaction was carried out at 70 °C for 5 h, then 5.5 g of zinc acetate was added into the system and the reaction continued for another 5 h. The product (PNZn-lignin) was washed a few times with distilled water and dried under reduced pressure until reaching constant weight at 60 °C. Composites of poly(butylene succinate) (PBS) containing both modified and unmodified lignin were obtained by melt compounding using the same equipment as before and with analogous conditions, with the exception of the temperature (120 °C). The addition of PNZn-lignin into the PBS matrix increased the thermal stability of the composite, and 10 wt.% of such filler significantly reduces the PHRR and THR of PBS. Moreover, the formation of charred residue was greatly improved, and the capability of forming a thick char layer on the surface of PBS contributes to the reduction of flammability and smoke formation [92].

In the work of Zong et al. [90], acetic acid lignin (AAL), obtained by acetic acid pulping and biobutanol lignin (BBL), being a by-product of biobutanol industries, was modified by grafting acrylate polymers onto their structure. The modification procedure was performed according to the previous report by the group, in which only BBL was modified by grafting methyl methacrylate side chains [89]. In the reported work, 1 g of AAL lignin was added into the mixture of calcium chloride dissolved in 20 mL of dimethyl sulfoxide and stirred for 20 min, then n-butyl methacrylate (BMA) and 1 mL of hydrogen peroxide were added. The mixture was stirred for another 5 min and heated to 50 °C. The termination of the reaction was achieved by the addition of a small amount of 4-methoxyphenol, and the resulting mixture was poured slowly into the diluted hydrochloric acid. The formed precipitate was filtered, washed with water, and finally dried in the oven at 60 °C for 24 under vacuum conditions to obtain AAL-g-PBMA. Similarly, as described above, lignin with grafted side chains of poly(methyl methacrylate) (PMMA), poly(benzyl methacrylate) (PBZMA), and poly(ethyl methacrylate) (PEMA)—AAL-g-PMMA, BBL-g-PBZMA, and BBL-g-PEMA, respectively—were obtained. All the mentioned materials were used in the fabrication of composites with PLA by melt blending at 185 °C with a 60 rpm rotor speed, with polyacrylate-grafted lignin content varying between 1 and 10 wt.%. The testing samples were obtained by hot-pressing the aforementioned composites at 30 MPa. Results of this study show successful grafting of acrylate monomers onto the used lignins with almost no homopolymerization occurring. The glass transition temperature of lignin-acrylate copolymers is higher than for acrylate homopolymers; moreover, the presence of polymeric side chains in both AAL and BBL lignin increases their thermal stability and hydrophobicity. As for the usage of lignin-acrylate copolymers in PLA composites, their incorporation enhanced the UV resistance and mechanical properties of the composites only at a low filler content [90].

Modification of LignoBoost kraft lignin using three different dihydroxybenzenes: catechol, resorcinol, and hydroquinone, was presented by Hoffmann et al. [93]. After melting 10 g of the chosen dihydroxybenzene, 5 g of lignin was introduced, and the mixture was treated with concentrated H_2_SO_4_ in the quantity of 0.1 mL per gram of lignin and stirred for 30 min at 180 °C. After cooling the reaction mixture and its dissolution in 150 mL of a 1:1 acetone:water solution, the product was precipitated by pouring the mixture into a 4-fold amount of 1 M H_2_SO_4_. After the precipitation, the product was separated with a filter crucible, washed with water, dried, and washed several times with diethyl ether. After drying in vacuo, the products—catechol (LBL_ct), resorcinol (LBL_rs), and hydroquinone-modified lignin (LBL_hq)—were stored in a desiccator over P_2_O_5_. As a result of the modification, lignins with a higher phenolic content and a more uniform structure were obtained. Moreover, a higher number of phenolic hydroxyl groups due to the modification increases the number of possible crosslinking areas and provides better metal adsorption properties for the modified lignin [93].

Lignin sulfonates were also obtained through sulfonation of Western Hemlock wood chips (W), and lignin sulfonates prepared from milled wood lignin (MWL) were fractionated by Sephadex gel chromatography in aqueous solution and then acetylated and methylated. The resulting acetyl lignin sulfonate methyl ester (ALSME) fractions were characterized by nuclear magnetic resonance spectroscopy and ALSME-MWL fractions were also characterized by chemical analyses. Gel permeation chromatography of ALSME fractions was carried out in methanol on Sephadex LH-20 and yielded further evidence indicating the separation in part of low molecular weight ALSME species, including one which was obtained in crystalline form [107].

Oiivares and co-workers [108] performed chemical modification of lignin and black liquor to improve the chemical reactivity. The following modifications were investigated: (a) methylolation of lignin as recommended by Dolenko and Clarke [109], (b) demethylation of lignin, and (c) separation of high molecular weight fractions of black liquor by ultrafiltration, followed by acid precipitation of lignin. Mechanical properties and water resistance were evaluated through testing particleboard panels manufactured with the obtained resins. The best properties were found for resin composed of 18.8% ultra-filtrated high molecular weight lignin, 22.9% phenol, and 58.3% formaldehyde, which had comparable properties with typical commercial resins prepared only with phenol and formaldehyde.

## 5. Physical Modification

Physical techniques of modification do not involve direct reactions between functional groups present in lignin and other compounds but employ physical processes which impart new, different properties to the modified material.

The work of Rao et al. [110] describes the impact of gamma irradiation on the properties of kraft lignin. In this paper, kraft lignin was subjected to gamma irradiation with doses of 30, 60, and 90 kGy using a Gamma Chamber. After the irradiation, lignin was analyzed with spectroscopic, thermal, and morphological methods to determine the changes in the properties. The SEM results show that irradiation caused the differences in the morphology, since untreated lignin displayed smooth, spherical particles of about 100 µm in size, whereas for the irradiated samples the size of particles gradually decreased. Since radiation exposure may lead to chain cleavage and a decreased molecular weight, changes in the glass transition temperature as well as temperatures of exothermic peaks are observed for the irradiated samples. Moreover, gamma irradiation greatly enhanced the radical scavenging ability of lignin [110].

**Figure 12:**
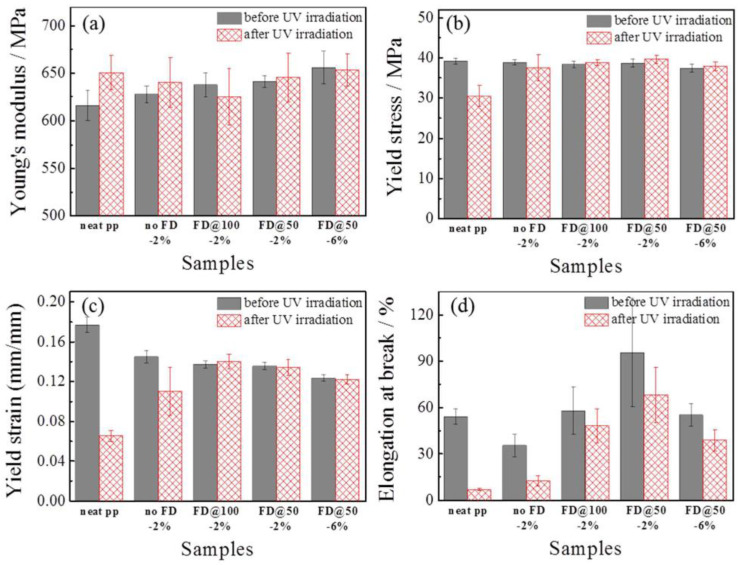
Tensile mechanical properties of the PP/lignin composites before and after UV irradiation. (**a**) Young’s modulus, (**b**) yield stress, (**c**) yield strain, and (**d**) elongation at break of neat PP, PP/lignin composites with 2 wt.% as-received lignin (no FD-2%), 2 wt.% freeze-dried lignin at a lignin concentration of 100 mg/mL (FD@100-2%), 2 wt.% freeze-dried lignin at a lignin concentration of 50 mg/mL (FD@50-2%), and 6% freeze-dried lignin at a lignin concentration of 50 mg/mL (FD@50-6%). Reproduced from [111], with the permission of the American Chemical Society.

In another development, alkali lignin was used to prepare colloidal solutions in deionized water with the concentrations of 50 and 100 mg of lignin per mL. To ensure proper dispersion, bottles with solutions were placed in an ultrasonic bath and then frozen using liquid nitrogen, followed by freeze-drying in vacuum conditions for 3 days. To prepare composites with PP matrix, untreated and freeze-dried lignins were mixed by melt compounding with polymer pellets in a Haake MiniLab twin-screw extruder at 190 °C. The results of this study show that for the freeze-dried lignin particles obtained from the 50 mg/mL solution, a sheet-like morphology was observed. Such structure enables the particles to easily break during the processing, leading to a more uniform filler dispersion in the composites with PP and a larger interfacial area of the particles in relation to the untreated lignin, which contains spherical- or irregular-shaped particles. The mechanical tests revealed that PP composites containing freeze-dried lignin display improved values of Young’s modulus and elongation at break, in comparison to composites with untreated lignin or pure PP. Moreover, tests regarding UV-induced and thermo-oxidative degradation indicate that sheet-like lignin acts as a free radical scavenger, slowing the aging process of PP composites [111] (Figure 12).

Lignin modified by the sorption of metal ions was prepared by El Zawawy and co-workers. Lignin was first isolated from the rice straw by alkaline pulping and the obtained black liquor was acidified by the dropwise addition of concentrated H_2_SO_4_ until pH 1.5. The resultant mixture was stirred continuously and heated at 100 °C in a water bath. The fraction containing precipitated lignin was cooled down to room temperature, filtered using a Buchner funnel, washed with hot water until neutral pH, and finally air-dried at ambient conditions. Isolated lignin was dissolved in 17.5 wt.% of NaOH solution and precipitated with H_2_SO_4_ using the same procedure. The precipitate was filtered, washed with hot water until obtaining a clear filtrate, and air-dried at ambient conditions. To prepare modified lignin materials, first, a lignin model compound—vanillin (V)—was subjected to modifications. Metal surface complexes were obtained by sorption of metal cations—Fe^3+^, Ni^2+^, and Co^2+^—present in solutions with a concentration of 2 × 10^−4^ mol/L. An analogous procedure was performed for the alkali lignin (AL) obtained in the previous step. Both modified and unmodified samples of AL and V were introduced at 5% loading into the polystyrene matrix to obtain composite materials via extrusion using a micro-compounder system at 130 °C for 10 min at 75 rpm. The resultant composites were molded into films of 0.5 mm thickness. Obtained samples were tested to determine the changes in the spectra which could be attributed to the formation of metal complexes. For both V and AL modified with cobalt ions, the UV spectra displayed the appearance of a new band at λmax = 258 nm, which confirmed the formation of the lignin-Co^2+^ complex. Those samples were chosen for further investigation, which revealed better miscibility with the matrix for vanillin and lignin metal complexes than for their unmodified counterparts. This is attributed to higher values of tensile strength for PS composites with complexed lignin and vanillin than other materials; however, the addition of any kind of filler to the PS matrix resulted in a decrease of the elongation at break [112].

The work by Souza Jr. et al. [113] describes the utilization of plasma treatment to obtain crosslinked coatings from lignin. Dispersions of soda lignin in the acetone:water mixture (9:1) were used to prepare films on different substrates using the spin-coating method. The samples were divided into the following groups: an untreated control group, two groups subjected to SF_6_ plasma treatment (15 and 30 min of exposure, respectively), and two groups irradiated with UV light (also 15 and 30 min of exposure). The results show that UV irradiation caused the formation of smooth coatings and contributed to the suppression of OH band intensity and the formation of C=O bonds, which imparted hydrophilic properties to the lignin surface. On the other hand, SF_6_ plasma treatment caused substantial changes in the surface structure, causing roughness and incorporation of CF_x_ groups, which caused the super-hydrophobicity of as-treated lignin. Such properties are an attractive asset, with the potential to be applied in self-cleaning, anti-fouling, and anti-corrosive materials [113].

In the recent research concerning the usage of physical methods of modification to enhance the properties of lignin (Table 12), improvement of the radical scavenging ability of lignin by employing gamma irradiation and freeze-drying seems to be in the spotlight [110,111]. Better mechanical properties were reported in the studies where lignin underwent modification via freeze-drying [111] and sorption of metal ions onto its surface [112]. UV irradiation caused enhanced hydrophilicity of the lignin, whereas plasma treatment resulted in obtaining the material with superhydrophobic properties [113]. However, using strong gamma irradiation may cause a decrease in the molecular weight of lignin due to the radiation-induced degradation processes [110].

## 6. Future Perspectives

Results of research works dedicated to chemical and physical modification of technical lignin show great potential for usage of this aromatic macromolecule of natural origin. Although in recent years most of the publications concentrated on lignin modification via esterification, there are also other approaches developed to yield new biomaterials with enhanced properties. Modified lignin is used as a filler in composite materials with conventional polymer matrices and biobased ones towards fabrication of environmentally friendly composite and hybrid materials, showing vast potential in, e.g., biomedical and environmental protection sectors. Recently, an increase of interest has been observed in utilizing bio-based polymers from renewable resources as an alternative solution to some of the conventional polymer applications, and it seems that future research will concentrate on materials and methods of modification suitable for addressing this issue. The current environmental concerns, such as carbon footprint, a decrease of generation of greenhouse gases, and obtaining materials and chemicals consistent with the principles of green chemistry, will also be addressed.

Chemical and physical modification of lignin makes it possible to achieve a positive enhancement of the intrinsic lignin properties or introduction of desirable features to the modified material. However, some drawbacks and challenges encountered during the modification experiments, as well as the appearance of undesirable worsening of products’ properties, have also been noticed. Another challenge related to the lignin application results from the chemical and biological nature of this polymer. Since the composition and the structure of lignin are heavily dependent on the plant source, special attention needs to be paid to standardizing lignin-based materials and assuring their reproducible properties. Moreover, the type of technical lignin used as a starting material influences the choice of possible modification methods, since some of the properties, namely solubility, are dependent on the lignin extraction process and may hinder some of the modification approaches. Although lignin already shows great potential in composites’ applications, there is still room for improvement and the need to search for new ways of possible functionalization of lignin.

## 7. Conclusions

Recent works on the chemical and physical modification of technical lignin show the great potential of this aromatic macromolecule of natural origin. Although most of the publications concern lignin modification via esterification, other modification routes are also explored. Modified lignin is currently vastly used as a filler in composite materials, with bio-based, biodegradable polymers showing potential in biomedical or environmental applications. Green materials’ developments cause an increase in interest in utilizing bio-based polymers from renewable resources, such as lignin, as an alternative solution to conventional polymeric materials. Future research will most likely concentrate on lignin-based organic–inorganic hybrid materials and composites with enhanced properties, as well as new methods of modification, both chemical and physical.

## Figures and Tables

**Table 1:** Abbreviations used in the review with their respective explanations.

Abbreviation	Explanation
**CHEMICALS**	
H_2_SO_4_	sulfuric acid
THF	tetrahydrofuran
NaOH	sodium hydroxide
DMF	dimethylformamide
TEC	triethyl citrate
ATBC	acetyl tributyl citrate
TCP	tricresyl phosphate
BMA	n-butyl methacrylate
DVB	divinylbenzene
HCl	hydrochloric acid
NH_4_OH	ammonia aqueous solution
KOH	potassium hydroxide
HFP	1,1,1,3,3,3,-hexafluoro-2-propanol
LBL	LignoBoost Lignin
HMAP	4-hydroxy-3-methoxyacetophenone
DETA	diethylenetriamine
APP	ammonium polyphosphate
EDC	N-(3-dimethylaminopropyl)-N’-ethylcarbodiimide hydrochloride
TDMSCl	tert-butyldimethylsilyl chloride
DVAPTS	γ-divinyl-3-aminopropyltriethoxysilane
PEI	poly(ethylene imine)
DEP	Diethyl phosphite
WP	wood powder
AAL	acetic acid lignin
BBL	biobutanol lignin
P_2_O_5_	phosphorus pentoxide
V	vanilin
**POLYMERS**	
LDPE	low-density polyethylene
HDPE	high-density polyethylene
PP	polypropylene
PS	polystyrene
PET	poly(ethylene terephthalate)
PU	polyurethanes
NR	natural rubber
BR	butadiene rubber
PBAT	poly(butylene adipate-co-terephthalate)
PBS	poly(butylene succinate)
PHA	polyhydroxyalkanoates
PLA	polylactide
PAN	polyacrylonitrile
PPC	poly(propylene carbonate)
PCLLA	poly(ε-caprolactone-co-lactide)
PCL	polycaprolactone
PLLA	poly(L-lactic acid)
PHB	poly(3-hydroxybutyrate)
WPU	waterborne polyurethanes
PVC	poly(vinyl chloride)
PMMA	poly(methyl methacrylate)
PBZMA	poly(benzyl methacrylate)
PEMA	poly(ethyl methacrylate)
**MATERIAL SAMPLES**	
CTA	cellulose triacetate
MALig	maleic anhydride-modified kraft lignin
CELig	dichloroethane-modified kraft lignin
CMLig	dichloromethane-modified kraft lignin
CBLig	chlorobenzene-modified kraft lignin
LCC	lignin-carbohydrate complex
PUFs	polyurethane foams
LPB	lignin-PHB copolymer
LPHC	random lignin-PHB-PCL copolymer
LPH+C	block copolymer of lignin with PHB and PCL
LPC+H	block copolymer of lignin with PCL and PHB
P-LBL	phenolated LignoBoost Lignin
PLCD	poly(ethylene imine)-anchored lignin composite
UM-Lig	urea-modified lignin
CLig	lignin modified with DVAPTS
PNZn-lignin	product of reaction of lignin with PEI, DEP, and zinc acetate
AAL-g-PBMA	acetic acid lignin grafted with side chains of poly(butyl methacrylate)
AAL-g-PMMA	acetic acid lignin grafted with side chains of poly(methyl methacrylate)
BBL-g-PBZMA	biobutanol lignin grafted with side chains of poly(benzyl methacrylate)
BBL-g-PEMA	biobutanol lignin grafted with side chains of poly(ethyl methacrylate)
LBL_ct	LignoBoost lignin modified with catechol
LBL_rs	LignoBoost lignin modified with resorcinol
LBL_hq	LignoBoost lignin modified with hydroquinone
**CHARACTERIZATION METHODS**	
SEM	Scanning electron microscopy
XPS	X-ray photoelectron spectroscopy
FTIR	Fourier-transform infrared spectroscopy
^1^H-NMR	Proton nuclear magnetic resonance
DSC	Differential scanning calorimetry
TGA	Thermogravimetric analysis
**PROPERTIES**	
LOI	Limiting oxygen index
PHRR	Peak heat release rate
THR	Total heat release

**Table 2:** Monolignol content in different plant sources. Reproduced from [12], with the permission of the American Chemical Society.

Monolignol	Conifer Wood	Broadleaf Wood	Grass
Sinapyl alcohol	0–1%	50–75%	25–50%
Coniferyl alcohol	90–95%	25–50%	25–50%
p-Coumaryl alcohol	0.5–3.4%	trace amounts	10–25%

**Table 3:** Typical properties of technical lignin. Reproduced from [9], with the permission of Elsevier.

Lignin Properties	Sulfur Lignins		Sulfur-Free Lignins	
	Kraft	Lignosulfonate	Soda	Organosolv
Raw material	Softwood Hardwood	Softwood Hardwood	Annual plants	Softwood Hardwood Annual plants
Solubility	Alkali Organic solvents	Water	Alkali	Wide range of organic solvents
Number-average molar mass (M_n_ g·mol^−1^)	1000–3000	15,000–50,000	800–3000	500–5000
Dispersity	2.5–3.5	6–8	2.5–3.5	1.5–2.5
T_g_ (°C)	140–150	130	140	90–110

**Table 4:** Modification methods of lignin and chemical agents.

Type of Modification	Chemical Reaction	Chemical Agent
Chemical modification	Esterification	Acyl chlorides
		Carboxylic anhydrides
		Carboxylic acids
		Lactones
	Alkylation and arylation	Chlorinated hydrocarbons
		Heterocyclic hydrocarbons
		Carboxylic acids
	Epoxidation	Epichlorohydrin derivatives
	Hydroxymethylation	Aldehydes
		Cyclic ethers
	Copolymerization	Anilines
		Lactides
		Lactones
	Amination	Amine compounds
	Silylation	Silica-containing agents
	Methylolation	Aldehydes
	Demethylation	Dichromate salt with aldehydes or organic acids
	Sulfonation	Aqueous solution of sulfur dioxide
	Alkoxylation	Cyclic ethers
	Hydrolysis	Hydrochloric acids, chlorosulfonic acid
	Other	Phosphorous compounds
		Acrylic compounds
		Ketones
		Benzenediols
Physical modification	-	Freeze-drying
		UV irradiation
		Gamma irradiation
		Plasma treatment
		Ultrasonic homogenization and sorption of compounds onto the surface

**Table 5:** Table summarizing the esterification process of lignin.

Type of Lignin	Modifying Agents	Conditions	Properties after Modification
Soda lignin	10-undecenoyl chloride, oleoyl chloride	65 °C, 46 h, no additional solvents or catalysts	higher UV-absorption capacity possibly better thermal stability improved mechanical properties
Kraft lignin	Acetic anhydride, propionic anhydride	85 °C	better dimensional stability reduced hygroscopicity improved resistance to microbial decay reduced mechanical properties
Soda lignin	Butyric anhydride	50 °C, overnight reaction, catalyst: 1-methylimidazole	increasing Young’s modulus decreasing the thermal degradation at low temperatures
Kraft lignin	H_2_SO_4_, γ-valerolactone, acetyl ketene, butyric anhydride	γ-valerolactone: solution of lignin in γ-valerolactone stirred at 140 °C for 5 h, after addition of acetyl ketene stirred at 90 °C for 1 h butyrated lignin: stirred at 120 °C for 24 h, catalyst: 1-methylimidazole	better interfacial compatibility higher tensile strength higher storage modulus better thermal stability higher rate of thermal degradation than PLA
Organosolv lignin	Oleic acid, lactic acid, butyric acid, butyrolactone	Lignin powder impregnated with 5 wt.% reagent solution, followed by cold plasma modification at 500 Hz, 50 W for 60 min	better compatibility better solubility better intermolecular interactions
Kraft lignin	Succinic anhydride	Dissolution of lignin in pyridine under sonication, followed by addition of succinic anhydride and stirring overnight at 70 °C	reduced crosslinking density enhanced mechanical properties
Kraft lignin	Maleic anhydride, dichloroethane	Maleic anhydride: lignin was added in small portions at 100 °C to molten maleic anhydride, then the reaction mixture was placed in a microwave oven (2.45 GHz) for 20 min Dichloroethane: lignin was mixed with an excess of dichloroethane, catalyst: anhydrous aluminum chloride, and refluxed for 20 min in a modified microwave oven	better miscibility better compatibility better solubility better intermolecular interactions possible comparable mechanical properties to PP
Alkali (kraft) lignin	Maleic anhydride	After dissolution of lignin in 20 wt.% aqueous NaOH solution and 1 h of stirring, maleic anhydride was added and the reaction continued for 4 h at 70 °C	improved miscibility between the matrix and the filler the shift of the glass transition temperature toward higher temperatures decrease in the value of elongation at break increase in tensile strength and Young’s modulus hindered the rate of microbial degradation
Alkali (kraft) lignin, dealkali lignin	Tung oil, acrylic acid, n-butyl methacrylate	Tung oil: 50–60 °C, 2 h, mechanical stirring, alkaline conditions Acrylic acid: reagents refluxed for 24 h, acidic conditions n-butyl methacrylate: 50 °C, 2 h, stirring, alkaline conditions	possible increase in the glass transition temperature decrease in moisture content partial depolymerization of lignin
Kraft lignin	Phthalic anhydride	120 °C, 3 h, continuous stirring, in presence of pyridine	tensile strength—values similar to LDPE lower content of lignin phthalate—quasi-brittle failure fracture surface higher content—brittle fracture surface higher content—lower weight loss, char formation

**Table 6:** Table summarizing alkylation and arylation processes of lignin.

Type of Lignin	Modifying Agents	Conditions	Properties after Modification
Kraft lignin	Dichloromethane, chlorobenzene	Dichloromethane: lignin and dichloromethane refluxed for 1 h Chlorobenzene: lignin mixed with chlorobenzene, 0.5 h in room temperature followed by reflux for 2 h at boiling point Both reaction used anhydrous aluminum chloride as a catalyst	better compatibility better thermal stability the shift of melting temperature toward lower temperatures better mechanical properties
Kraft lignin	γ-butyrolactone, tetrahydrofuran	γ-butyrolactone: 200 °C, 1 h, 1 wt.% of H_2_SO_4_ as a catalyst, followed by another 1 h at 250 °C THF: 30 min at 50 °C, then 30 min at 100 °C and 1 h at 150 °C, 1 wt.% of H_2_SO_4_ as a catalyst	decreased tensile strength decreased Young’s modulus increased strain at break
Kraft lignin	Lactic acid, tetrahydrofuran	Lactic acid: low molecular weight PLA obtained by condensation was combined with lignin and 2 wt.% of lactic acid (catalyst) and stirred for 2 h at 180 °C THF: 30 min at 100 °C, then 2 h at 150 °C, 1 wt.% of H_2_SO_4_ as a catalyst	deterioration of mechanical properties with the increased filler content

**Table 7:** Table summarizing the epoxidation process of lignin.

Type of Lignin	Modifying Agents	Conditions	Properties after Modification
Organosolv lignin	Epichlorohydrin	Depolymerized lignin was reacted with epichlorohydrin under alkaline conditions for 3 or 5 h, at 50, 70, and 90 °C, with varying epichlorohydrin/lignin molar ratios: 1, 2, 4, and 10	homogeneous structure better mechanical properties better value of compressive stress better stable relative deformation
Alkali (kraft) lignin, hydroxy-methylated kraft lignin	Epichlorohydrin	Epoxidation: 50, 70, or 90 °C, and 3, 5, or 7 h; lignin:NaOH (*w*/*w*) ratio, 1:3 or 1:6, lignin:epichlorohydrin (*w*/*w*) ratio, 1:10	
Alkali (kraft) lignin	Epichlorohydrin	After dissolution of lignin in the mixture of 5 wt.% NaOH and 37% formaldehyde solution, the solution was stirred at 80 °C for 2 h, followed by the addition of epoxy chloropropane, and then stirred for 4 h at 80 °C	

**Table 8:** Table summarizing the hydroxymethylation reaction of lignin.

Type of Lignin	Modifying Agents	Conditions	Properties after Modification
Alkali (kraft) lignin	Formaldehyde	After dissolution of lignin in NaOH solution, formaldehyde (2.5:1 molar ratio of formaldehyde:lignin) was added, followed by heating to 50 °C and adjusting pH with HCl	poor miscibility filler agglomeration heterogeneous structure
Alkali (kraft) lignin	Formaldehyde	After dissolution of lignin in the mixture of distilled water, 5 wt.% of NaOH, and a 37% formaldehyde solution, the solution was stirred at 80 °C for 4 h, followed by the pH adjustment with HCl	
Kraft lignin	Formaldehyde	Mixture of lignin with distilled water stirred for 2 h at room temperature, followed by the addition of NaOH and NH_4_OH as a catalyst and shaking of the whole mixture for 2 h. After the introduction of 37 wt.% of formaldehyde solution, the reaction was carried out for 4 h at 85 °C and then ended by pH adjustment with HCl	improved the processability of the rubber increased the crosslinking density increased the curing rate of the rubber compounds improved mechanical properties
Kraft lignin	Formaldehyde	Mixture of lignin with distilled water stirred for 2 h at room temperature, followed by heating the mixture to a set temperature (50, 72.5, 95 °C) and addition of NaOH and NH_4_OH as a catalyst, and stirring the mixture for 2 h. After the introduction of the 37 wt.% formaldehyde solution (lignin:aldehyde ratio between 1:2 and 2:1), the reaction was carried out for another 2 h and ended by pH adjustment with HCl	increased tensile strength increased Young’s modulus increased thermal stability
Kraft lignin	Formaldehyde	Lignin dispersion in deionized water with pH adjusted to 12 by the addition of NaOH was heated to 90 °C, followed by the dropwise addition of formaldehyde, and reacted for 3 h	
Kraft lignin	Propylene oxide	Lignin was dispersed in deionized water and the pH was adjusted to 12 by the addition of NaOH. Then, propylene oxide was added dropwise and the reaction continued at 30 °C for 10 h	improved compatibility with the matrix increased tensile strength increased Young’s modulus increased thermal stability ability of microbial degradation
Kraft lignin, soda lignin, Organosolv lignin	Propylene oxide	Set amounts of lignin/propylene oxide (*w*/*v*)—10/90, 20/80, 30/70, and 40/60—and KOH as a catalyst were placed in a reactor and heated under stirring to 160 °C	

**Table 9:** Table summarizing copolymerization reactions of lignin.

Type of Lignin	Modifying Agents	Conditions	Properties after Modification
Alkali (kraft) lignin	ε-caprolactone, L-lactide	Lignin, ε-caprolactone, and L-lactide with set mass ratios (2:2.4:5.6, 2:4:4, 2:5.6:2.4) and 0.5 wt.% (of monomer) of tin(II) 2-ethylhexanoate as a catalyst were placed in a flask and stirred at 130 °C for 24 h, nitrogen atmosphere	good adsorption capacity increased their rate of degradation biocompatible non-cytotoxic
Kraft lignin	L-lactide	Lignin and L-lactide in set mass ratios (10/90, 20/80, 30/70, 40/60, 50/50) were placed in a reactor and reacted for 3.5 h at 130 °C in the presence of triazabicyclodecene as a catalyst. Reaction ended by the addition of acetic acid solution in dichloromethane	UV-blocking properties increased tensile strength possible increased tensile modulus
Alkali (kraft) lignin	β-butyrolactone, ε-caprolactone	LPB: lignin and β-butyrolactone (weight ratio 2:8) and 5 wt.% (of monomer) of tin(II) 2-ethylhexanoate as a catalyst were placed in a flask and stirred at 350 rpm, at 130 °C for 4 h, nitrogen atmosphere LPHC: lignin reacted with both β-butyrolactone and ε-caprolactone (weight ratio 2:4:4) under analogous conditions for 8 h LPH+C/LPC+H: lignin reacted first with β-butyrolactone or ε-caprolactone (weight ratio 2:4:4) for 4 h, followed by the addition of the latter monomer and continuation for another 4 h	possible decreased tensile strength possible decreased Young’s modulus possible decreased elongation at break better stability to degradation non-cytotoxicity
Alkali (kraft) lignin	β-butyrolactone	Lignin and β-butyrolactone (weight ratio 2:8) as well as tin(II) 2-ethylhexanoate (catalyst) were reacted at 130 °C for 24 h, under nitrogen atmosphere	increased tensile strength increased stiffness increased elongation antioxidant activity biocompatibility

**Table 10:** Table summarizing the amination reaction of lignin.

Type of Lignin	Modifying Agents	Conditions	Properties after Modification
Kraft lignin, alkali lignin	DETA, formaldehyde	Lignin and DETA were dissolved in distilled water, and after the adjustment of pH to alkaline, formaldehyde was added at room temperature while stirring and reaction continued after heating to 50 °C for 4 h	improved mechanical properties anti-aging performance better thermal stability
Alkaline lignin	Urea, formaldehyde	Lignin dissolved in NaOH aqueous solution was mixed with urea and formaldehyde, then refluxed at 70 °C for 10 h under mechanical stirring	decreased flammability improved the thermal stability improved char formation deterioration of mechanical properties
Kraft lignin	Glycine, sodium acetate trihydrate, formaldehyde	Lignin was mixed with glycine and sodium acetate trihydrate solution in acetic acid, then formaldehyde was added at 50 °C	improved mechanical properties increase in elasticity negative influence on the cytotoxicity

**Table 11:** Table summarizing the silylation reaction of lignin.

Type of Lignin	Modifying Agents	Conditions	Properties after Modification
Kraft lignin	Silica	Reagents were combined via grinding in a planetary ball mill for 12 h with intervals of 15 min, after which the direction of rotation was changed. Every 2 h, the mill was switched off for 5 min to avoid overheating	decreased Young’s modulus decreased tensile strength increased Vicat temperature better thermal stability
Kraft lignin	Silica	Reagents were combined using a grinder mortar for 2 h, with a 2 min break after each 30 min of grinding to avoid overheating	high nucleation ability increased thermal stability
Soda lignin	Tert-butyldimethylsilyl chloride	TDMSCl and imidazole were added to lignin dissolved in DMF and the reaction was carried out at room temperature for up to 18 h	better thermal stability better solubility in organic solvents improved compatibility with the matrix
Soda lignin	γ-divinyl-3-aminopropyl-triethoxysilane	After dissolution of DVAPTS in a 5:95 (*v*/*v*) mixture of water and ethanol, it was combined with soda lignin and stirred	better thermal stability decreased flammability char formation better thermal stability good mechanical properties

**Table 12:** Table summarizing physical modification processes of lignin.

Type of Lignin	Conditions	Properties after Modification
Alkali lignin	Colloidal solutions of lignin (50 and 100 mg/mL) in deionized water were dispersed in an ultrasonic bath, frozen with liquid nitrogen, and freeze-dried	improved Young’s modulus improved elongation at break better thermal stability
Alkali lignin, vanilin	Both alkali lignin and vanillin underwent sorption of metal cations—Fe^3+^, Ni^2+^, and Co^2+^—to form metal surface complexes	better miscibility with matrix higher values of tensile strength decreased the elongation at brea
Soda lignin	Using the spin-coating method, thin films from dispersions of soda lignin in an acetone:water mixture (9:1) were prepared. Substrates were divided into: untreated control group, SF_6_-plasma treated group (15 and 30 min of exposure), and UV-light irradiated group (15 and 30 min of exposure)	superhydrophobic properties self-cleaning properties anti-fouling properties anti-corrosive properties

## Data Availability

The data are contained within the article.
